# Early Determinants of Work Disability in an International Perspective

**DOI:** 10.1007/s13524-020-00902-7

**Published:** 2020-08-25

**Authors:** Axel Börsch-Supan, Tabea Bucher-Koenen, Felizia Hanemann

**Affiliations:** 1 Munich Center for the Economics of Aging (MEA) at the Max Planck Institute for Social Law and Social Policy, Amalienstrasse 33, D-80799, Munich, Germany; 2 Department of Economics and Business, Technical University of Munich (TUM), Munich, Germany; 3 National Bureau of Economic Research (NBER), Cambridge, MA USA; 4 ZEW – Leibniz Center for European Economic Research, Mannheim, Germany; 5 University of Mannheim, Mannheim, Germany

**Keywords:** Social security and public pensions, Work disability, Disability insurance, International comparisons, Life histories

## Abstract

This study explores the interrelated roles of health and welfare state policies in the decision to take up disability insurance (DI) benefits due to work disability (WD), defined as the (partial) inability to engage in gainful employment as a result of physical or mental illness. We exploit the large international variation of health, self-reported WD, and the uptake of DI benefits in the United States and Europe using a harmonized data set with life history information assembled from SHARE, ELSA, and HRS. We find that the mismatch between WD and DI benefit receipt varies greatly across countries. Objective health explains a substantial share of the within-country variation in DI, but this is not the case for the variation across countries. Rather, most of the variation between countries and the mismatches are explained by differences in DI policies.

**Electronic supplementary material:**

The online version of this article (10.1007/s13524-020-00902-7) contains supplementary material, which is available to authorized users.

## Introduction

Work disability (WD) is the (partial) inability to engage in gainful employment due to physical or mental illness, resulting in early retirement and/or uptake of disability insurance benefits (Loisel and Anema 2014). Disability insurance (DI) is a substantial part of public social expenditures and an important part of the social safety net of all developed countries (OECD 2003, 2010). This study explores the relation between WD and DI from an international perspective.

**Fig. 1:**
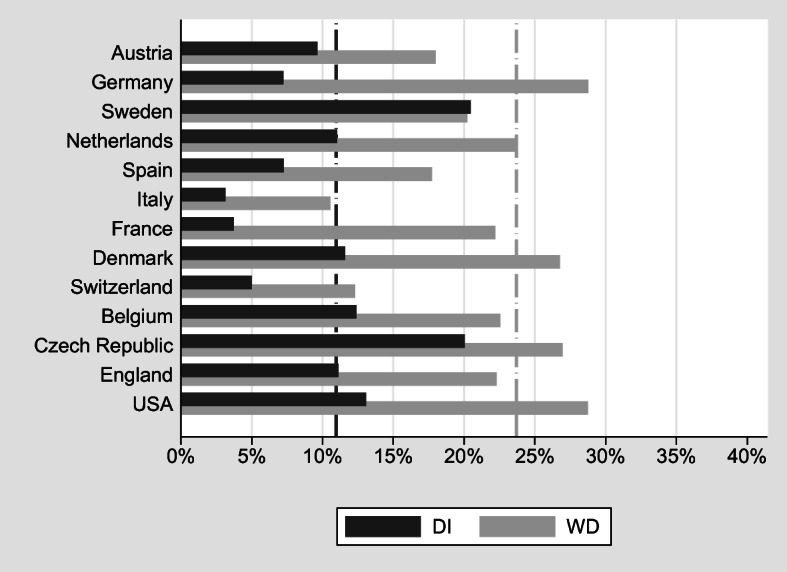
WD and DI receipt in Europe and the United States among individuals aged 50–65. *Source:* Own calculations based on weighted data from SHARE Wave 5, ELSA Wave 6, and HRS Wave 11.

The design of WD insurance systems is a challenging task for policy-makers (Autor and Duggan 2003, 2006, 2010; Burkhauser et al. 1999; de Jong et al. 2011; Haveman and Wolfe 2000). Like almost all elements of modern social security systems, DI faces a trade-off (Aarts et al. 1996; Autor et al. 2016; Banks et al. 2004; Croda and Skinner 2009; Diamond and Sheshinski 1995). On the one hand, DI is a welcome and necessary part of the social safety net: it prevents income losses for those who lose their ability to work before they become eligible for old-age pensions. On the other hand, DI may be (mis-)used as an early retirement route even if the ability to work is not limited. Both self-reported WD and DI uptake vary substantially among European countries and the United States (Fig. 1), based on data on individuals aged 50–65 from the Survey of Health, Ageing and Retirement in Europe (SHARE), the English Longitudinal Study on Ageing (ELSA), and the U.S. Health and Retirement Study (HRS).^1^

Whereas approximately 23% of these respondents report suffering from a disability that limits their working capacity, this percentage is much lower in Italy (roughly 10%) and almost three times that size in Germany and the United States. Similarly, the share of individuals receiving DI benefits ranges from approximately 3% to 5% in Italy, France, and Switzerland to about 20% in Sweden and the Czech Republic. In almost all countries, more individuals report WD than DI. However, there are marked cross-national differences in the relative size of the WD and DI populations. In Sweden, these populations are about equal; in France, about five times as many individuals report WD as receiving DI.

The aim of this study is to shed light on the interrelated roles of health and welfare state policies in the decision to take up DI due to WD. Regarding health, we especially focus on health over the entire life course. The key idea is to exploit the large variation of the potential causes for reporting WD and/or receiving DI benefits within and between countries.

A first and obvious potential cause for reporting WD and/or receiving DI benefits is current health. Heterogeneity of mortality and morbidity in Europe is large, both across and within countries. Life expectancy at birth for women in the EU varies between 86.3 years in Spain and 78.5 years in Bulgaria, and that for men varies between 81 years in Italy and 69.2 years in Lithuania (Eurostat 2016a). Although Swedish and Italian men have similar life expectancy (age 80.6 and 81, respectively), Swedish men spend more than five additional years in good health than their Italian counterparts: the gap in healthy life expectancy is 73.0 versus 67.6 years (Eurostat 2016b). Health varies strongly by income and other socioeconomic characteristics (European Union 2014). Health is more heterogeneous in the United States, Germany, and the Mediterranean countries than in Scandinavia (Avendano et al. 2009).

Second, ample evidence shows that good health in later life emerges from a person’s biological makeup, behavior, lifestyle, environmental and occupational conditions, health care interventions, and a multitude of interactions among these factors across the entire life span. An important insight of recent research is that these interactions manifest their effects starting very early in life and then accumulate in positive and negative feedback cycles over the entire life course (Conti and Heckman 2013; Power and Kuh 2006). Life course factors are therefore a second group of potential causes for reporting WD and/or receiving DI benefits.

Third, welfare-state policies, especially the design of the pension and DI systems, have been shown in the country studies edited by Gruber and Wise (1999, 2004) and Wise (2012, 2015) to create strong incentives on individuals’ labor market and retirement behavior. Thus, differences in policies are also likely to explain the large international variation in DI uptake rates. Burkhauser et al. (1999) studied the differences in work patterns between the Netherlands and the United States and showed that the generous retirement, disability, and unemployment benefits in the Netherlands at that time provided strong incentives for early withdrawal from the workforce. These incentives are more likely to explain the differences in the labor force participation rates among persons aged 50+ than differences in underlying health.

This article expands our research on early retirement and disability insurance in Europe and the United States (Börsch-Supan 2005, 2010, 2011; Börsch-Supan and Jürges 2012; Börsch-Supan and Roth 2011; Börsch-Supan and Schnabel 1998; Börsch-Supan et al. 2004) in four important respects. First, we systematically juxtapose WD with the uptake of DI. We find systematic international differences in the match between WD and DI.

Second, we stress the importance of life course events. We constructed an internationally harmonized data set assembled from the HRS, ELSA, and SHARE, with particular attention to lifetime health and other lifetime circumstances using the life history data from SHARE and ELSA plus comparable early childhood and life course data from HRS. We find that health problems experienced over the life course even as early as childhood are important drivers of later-life working capacity and the need to rely on DI benefits.

Third, there have been incisive reforms to the DI systems in many of the countries analyzed in earlier studies, and most significantly reduced the generosity of DI. In contrast to earlier studies based on cross-sectional data, we are able to exploit these policy changes thanks to our life history data. We can match the policy environment at the point in time when DI benefits were first received. Although the most striking international differences in DI generosity have been abolished, we still find a strong response of DI uptake to DI generosity that is identifiable even on the individual level.

Finally, we take account of measurement issues, potential biases, and reverse causality. Self-reported WD may be biased toward worse health outcomes given that the respondent may feel urged to justify enrollment in DI in spite of a good health status (Bound 1991; Dwyer et al. 2003; Kerkhofs and Lindeboom 1995). In turn, self-reports may also be positively biased because of accommodation (Hill et al. 2016). Self-reported general health is subject to similar measurement errors (Butler et al. 1987) and reporting biases (Benitez-Silva et al. 2000; Dwyer and Mitchell 1999). We address these by including more objectively measured health indicators included in SHARE, ELSA, and HRS: grip strength for upper-body physical health; EURO-D for depression; the sum of immediate and delayed word recall for memory abilities; and the number of limitations in the (instrumental) activities of daily living (ADL, IADL), which measure functional health. Nevertheless, we are careful in making causal attributions. To address reverse-causality problems, we exploit information about life health, made possible by our life history data. These variables measure health at childhood as well as episodes of ill health during the entire life course, allowing us to pick up health problems that occur well before the onset of WD and DI receipt and that are thus predetermined in the second-stage regression. We then use lifetime and objective health measures to predict WD and use these predictions in our comparisons with DI benefit receipt rather than self-reported WD.

## Analytical Framework

Although the cross-national differences visible in Fig. 1 are impressive at first sight, they are not straightforward to interpret. First, WD is self-reported and may depend on factors other than the objective health (**OBJH**) measures used for granting DI benefits. Second, WD and DI receipt are measured on very different scales.

WD is influenced by the *subjective assessment* (*SUBJ*) of the individual’s objective health. Moreover, WD may be subject to justification bias when the individual has applied for DI. The former is measurable, but the latter—for example, psychological factors determining the extent of the bias—are not and will enter as noise in the regression. WD may also depend on life circumstances, such as the age of the respondent, the availability of help by spouse and family, and the general environment in which the individual is living. We measure this environment by a set of demographic variables (**ENV**). Finally, WD is likely influenced by factors that occurred earlier in life, such as childhood conditions, frequent job changes, and health events earlier in life (**LIFE**). Because we measure WD as a binary indicator, we may write these relations as:

1 $$ WD=1\ \mathrm{if}\ \mathbf{OBJH}<\mathrm{threshold}\left( SUBJ,\mathbf{ENV},\mathbf{LIFE}\right). $$

Individuals self-report as work-disabled if their objective health is worse than a threshold that depends on their current environment, earlier life circumstances, and their subjective assessments of their health. This is equivalent to a probit regression:

2 $$ \mathrm{prob}\left( WD=1\right)=\Phi \left({\upsigma}_1\times SUBJ+{\upalpha}_1\times \mathbf{OBJH}+{\upbeta}_1\times \mathbf{ENV}+{\upgamma}_1\times \mathbf{LIFE}\right), $$

where Φ denotes the cumulative distribution function of the normal distribution.

The receipt of DI benefits depends on the severity of the WD as measured by the DI system. We denote this as $\hat{WD}$, which differs from WD: WD is influenced by the subjective factors *SUBJ*, but we postulate that the DI system uses objective criteria to measure $\hat{WD}$.

We do not observe $\hat{WD}$. As a proxy, we estimate a first-stage probit regression of WD on a set of objective health measures and the exogenous variables in **ENV**:

3 $$ \mathrm{prob}\left( WD=1\right)=\Phi \left({\upalpha}_2\times \mathbf{OBJH}+{\upbeta}_2\times \mathbf{ENV}+{\upgamma}_2\times \mathbf{LIFE}\right), $$

and we use the predicted value as a regressor for the second stage:

4 $$ \hat{WD}={\hat{\upalpha}}_2\times \mathbf{OBJH}+{\hat{\upbeta}}_2\times \mathbf{ENV}+{\hat{\upgamma}}_2\times \mathbf{LIFE}. $$

The social security system’s decision whether to grant DI benefits will depend on the prevailing policy rules (**POL**) that govern the generosity of DI benefits, the stringency of the application process, labor market policies, rehabilitation measures, and so on. DI benefit receipt may also be dependent on the life circumstances measured by **ENV**. Hence, we postulate

5 $$ \mathrm{DI}=1\ \mathrm{if}\ \hat{WD}>\mathrm{threshold}\left(\mathbf{POL},\mathbf{ENV}\right). $$

This is equivalent to a probit regression, our second-stage regression equation:

6 $$ \mathrm{Prob}\left(\mathrm{DI}=1\right)=\Phi \left({\uppi}_3\times \mathbf{POL}+{\upalpha}_3\times \hat{WD}+{\upbeta}_3\times \mathbf{ENV}\right). $$

In an ideal DI system, in which we are able to sort all individuals by $\hat{WD}$ from perfectly healthy to completely unable to work, DI recipients are therefore the upper *x*% of the $\hat{WD}$ distribution, where *x* is determined by the DI policy regime.

The regression Eqs. (2), (3), and (6) exploit both within-country and between-country variation in WD and DI benefit receipt because they will be estimated using pooled data from individuals in all countries.

The regressions will shed light on the role of life course health and other life course variables, providing insight, for example, on which lifetime factors contribute to whether people suffer from limitations on their earnings capacity later in life and have to rely on DI receipt. We assess how much of the total variation in WD and DI benefit receipt at the individual level is explained by the different sets of variables.

Second, we use the predicted $\hat{WD}$ to see how it matches DI benefit receipt—that is, whether individuals with an objectively measured $\hat{WD}$ actually receive help through DI on the one hand, and whether DI is not wasted on individuals without an objectively measured $\hat{WD}$ on the other hand. We use $\hat{WD}$ to assess the match quality of a DI system and compare this across countries.

Finally, we exploit our regression results to perform counterfactual simulations that set some of the explanatory variables to the average across countries. This helps us understand whether differences in the demographic structure, health, or DI policy characteristics can explain the large differences in the level of WD and DI benefit receipt between countries shown in Fig. 1.

## Data

### SHARE, ELSA, and HRS

The research presented here, based on data from a large set of countries, is possible because of the strict harmonization of variables in three sister studies on aging: SHARE, HRS, and ELSA. SHARE is a pan-European data set designed to analyze the process of population aging using cross-national comparisons within Europe and among Europe, the United States, and Asia (Börsch-Supan et al. 2013). SHARE is modeled closely after the U.S. HRS (Juster and Suzman 1995), which was the first survey of this kind, and the ELSA (Marmot et al. 2003), which followed the lead of the HRS. Harmonization allows for cross-country comparisons in cultures, living conditions, and policy approaches among Europe, England, and the United States if the information is sufficiently harmonized (King et al. 2004; National Research Council 2001). The potential of combining these data sets has not yet been fully exploited. Our harmonization efforts involve extensive data manipulation because of the often subtle differences in variable definitions across the three data sets. The data sets and the exact harmonization procedures are described in detail in section B of the online appendix.

We use the following waves of data: Wave 11 of the HRS, collected in 2012/2013; Wave 6 of the ELSA, collected in 2012/2013; and Wave 5 of SHARE, collected in 2013. For some variables, such as marital status and education, we merge information from previous waves (see Table B3 in the online appendix for details). A key feature of our harmonized data set is the availability of retrospective life history data about onset of WD, receipt of DI benefits, episodes of bad health, and other events that may explain WD and the receipt of DI benefits. Some of this information is available in the regular surveys. For some additional life history variables, we add information from SHARE Wave 3, ELSA Wave 3, and similar questions in HRS.^2^ Given the combination of data sets, we include 13 countries in most of our analyses: Austria, Germany, Sweden, the Netherlands, Spain, Italy, France, Denmark, Switzerland, Belgium, the Czech Republic, England, and the United States.

We restrict our analysis to individuals in an age range in which disability insurance occurs most frequently. Because of the age focus of all three studies, age 50 serves as the lower age bound in our analysis. In most countries, disability insurance benefits are automatically converted into old-age pension benefits; thus, our upper-age bound is the country-specific statutory retirement age.^3^ The upper-age bound ranges between 60 years and 66 years for some cohorts in the United States.

SHARE Wave 5 covers 20,428 individuals within this age range. ELSA includes 11,585 individuals and HRS covers 3,751 individuals. We keep only those individuals who have at least one job in their employment history (*N* = 32,929). After deleting observations with missing information for the dependent variables or the main health indicators, the remaining sample consists of 29,571 observations in total. The number of observations included in our regressions varies depending on the included control variables; in particular, some of the life course indicators are available only for subsamples.

### Variables

#### Dependent Variables (WD, DI)

We use two dependent variables: self-rated work disability (*WD*) and the receipt of disability benefits (*DI*). *WD* captures the self-assessed work disability based on the question, “Do you have any health problem or disability that limits the kind or amount of paid work you can do?” The second dependent variable, *DI*, is defined as receiving disability insurance benefits or not. Disability insurance is defined as all branches of publicly financed insurances providing compensation in case of the loss of the ability to perform gainful employment (see Table A3 in the online appendix for country-specific details). Both variables are binary. We observe 6,713 individuals (22.7%) who report WD and 3,033 individuals (10.3%) who receive DI benefits.^4^ The correlation between the two variables is high: about 80% of those with a DI report a health problem that limits their work capacity, and only about 16.2% of those not receiving DI benefits report such limitations. On the other hand, 36% of those with a health problem receive DI benefits, whereas only 2.7% of those without health problems receive DI benefits.

Our analytical framework suggests individual-level and country-level variables, described in the following subsections.

#### **Objective Health (OBJH)**

We use a broad set of objectively reported health measures. Functional health is measured by the number of limitations with activities of daily living (ADLs) and instrumental ADLs (IADLs). To account for a person’s mental well-being, we construct the EURO-D depression index based on the number of reported depressive symptoms in SHARE.^5^ We complement these health measures with information from the physical test measuring the maximal grip strength of a person. Grip strength is our most objective measure of health because the task is performed during the interview. It reflects the overall muscle status of the respondent and has been linked to mortality in previous research (see, e.g., Gale et al. 2007). We impute missing values for grip strength by setting them to 0, implying that the missing values originate from situations where persons are not able to perform the grip strength test because of frailty. We add an additional flag variable to control for these imputed values. Further, we include a cognition measure based on a verbal learning and recall test performed during the interview.

#### **Subjective Health (SUBJ)**

For comparison purposes, we also use the respondent’s self-reported health status rated on a categorical five-point scale from excellent (1) to poor (5). Self-reported health is among the most common measures used in public health surveys; it captures various physical, emotional, and social aspects of health and well-being, and has been found to predict mortality (see, e.g., Idler and Benyamini 1997; Jylhä 2009).

#### **Family and Social Environment (ENV)**

We proxy life circumstances by a set of demographic characteristics, education, and the individual’s employment history. We use gender and the respondent’s age at the time of the interview. Current marital status is categorized as married, divorced, widowed, or single.^6^ We include three categories of the highest educational attainment based on the International Standard Classification of Education (ISCED) coding (low education (0–2), medium education (3–4), and high education (5–6)).

#### **Life Course Events (LIFE)**

We measure childhood and adult health and economic events as a set of life course variables. We create the sum of all childhood illnesses the respondents had until they were 16 years old, covering infectious diseases, asthma, respiratory diseases, allergies, headaches, epilepsy, psychological problems, diabetes, heart problems, cancer, fractures, and chronic ear problems. The variable *adulthood diseases* is created accordingly and contains the sum of illnesses since age 16, including back pain, arthritis, osteoporosis, angina, heart diseases, diabetes, stroke, asthma, respiratory problems, headaches, cancer, psychiatric problems, fatigue, allergies, eyesight problems, and infectious diseases.

In addition, we use variables from the life histories in ELSA and SHARE to describe each respondent’s employment history. We construct the number of jobs during the work history by summing the employment spells (start and end of job). We also consider the situation between different employment spells and count all times of being sick or disabled as the number of working gaps. We further take into account whether the respondent had periods of ill health or disability that lasted for more than a year. Moreover, the socioeconomic status during childhood is measured by the number of books and the number of rooms in the accommodation at age 10. These variables are not available for those respondents in SHARE and ELSA who did not participate in the life history interviews, and are not available at all in the HRS.

Table 1 presents the summary statistics and some basic correlations. Further details can be found in Table B1 in the online appendix.

**Table 1:** Summary statistics and basic correlations (%)

	Categories	Share of Total Sample	WD = 0	WD = 1	DI = 0	DI = 1
DI	No DI	89.74	83.82	16.18		
	Receiving DI	10.26	20.28	79.72		
WD	No WD	77.30			97.31	2.69
	Reporting WD	22.70			63.98	36.02
Age	50–55	32.44	79.73	20.27	91.12	8.88
	56–60	39.96	77.66	22.34	89.59	10.41
	61–66	27.60	73.92	26.08	88.35	11.65
Gender	Male	46.37	78.29	21.71	89.81	10.19
	Female	53.63	76.44	23.56	89.68	10.32
Education	Low	24.51	72.06	27.94	85.86	14.14
	Medium	43.37	75.66	24.34	89.10	10.90
	High	30.18	83.93	16.07	93.90	6.10
Marital Status	Single	8.96	71.16	28.84	83.01	16.99
	Married	72.76	80.04	19.96	92.04	7.96
	Divorced	13.59	69.89	30.11	84.15	15.85
	Widowed	4.69	67.94	32.06	83.14	16.86
Number of Jobs	1–2	24.99	74.57	25.43	88.55	11.45
	3–4	13.78	77.81	22.19	90.45	9.55
	5–6	5.60	74.15	25.85	88.29	11.71
	>7	2.68	77.30	22.70	88.78	11.22
Self-reported Health	Excellent	12.49	96.37	3.63	97.62	2.38
	Very good	26.89	93.10	6.90	97.01	2.99
	Good	36.16	82.32	17.68	93.04	6.96
	Fair	18.63	50.58	49.42	77.90	22.10
	Poor	5.82	17.71	82.29	56.74	43.26
Number of Limitations, IADL	0	91.26	81.37	18.63	92.24	7.76
	1	5.87	43.52	56.48	70.12	29.88
	2	1.47	18.12	81.88	57.34	42.66
	>3	1.39	15.82	84.18	42.82	57.18
Number of Limitations, ADL	0	91.71	82.06	17.94	92.31	7.69
	1	4.44	33.64	66.36	68.72	31.28
	2	1.75	19.31	80.69	58.69	41.31
	>3	2.09	9.69	90.31	47.98	52.02
Grip Strength	0–20	4.16	53.46	46.54	76.81	23.19
	20–50	46.02	78.83	21.17	90.48	9.52
	40–60	27.64	82.25	17.75	91.99	8.01
	>60	2.06	86.56	13.44	94.43	5.57
EURO-D	0	22.96	91.99	8.01	96.47	3.53
	1–2	45.00	82.84	17.16	92.75	7.25
	3–4	19.33	66.29	33.71	84.55	15.45
	5–6	8.90	52.15	47.85	77.14	22.86
	>7	3.80	37.90	62.10	69.48	30.52
Recall Abilities	0–5	6.29	63.33	36.67	80.54	19.46
	6–10	41.25	74.23	25.77	87.70	12.30
	11–15	45.41	80.88	19.12	92.18	7.82
	16–20	7.05	84.61	15.39	94.20	5.80
Childhood Illnesses	0	14.16	80.18	19.82	92.86	7.14
	1–2	77.71	78.43	21.57	90.10	9.90
	3–4	7.36	63.59	36.41	82.25	17.75
	>5	0.78	41.30	58.70	68.26	31.74
Adulthood Illnesses	0	45.37	88.98	11.02	95.21	4.79
	1–2	43.96	73.67	26.33	88.71	11.29
	3–4	8.98	46.72	53.28	73.83	26.17
	>5	1.69	20.60	79.40	54.60	45.40

*Source:* Own calculations based on weighted data from SHARE Wave 5, ELSA Wave 6, and HRS Wave 11.

#### **Policy Indicators (POL)**

Finally, we merge country- and time-specific disability indicators to our microdata. They are provided by the OECD (2003, 2010) and measure the generosity of benefits in different DI systems on the basis of the following five characteristics: (1) coverage (ranging from the total population to employees only); (2) minimum disability level (lower bound ranging from 0% to 86%); (3) maximum benefit level (in terms of replacement rate (RR), ranging from <50% to ≥75%), (4) medical assessment (ranging from treating doctor only to teams of insurance doctors); and (5) vocational assessment (ranging from strict own-occupation assessment to all jobs available). Each indicator is measured according to a predefined scale ranging from 0 points (restrictive) to 5 points (generous). The sum of the indicators is used as covariate in the regression analyses to account for country differences in the generosity of DI benefit systems.^7^ The indicators are available for three points in time: around 1985, 2003, and 2007 (see Table A1, online appendix). We match the year of first DI benefit receipt of our individuals with these three periods to approximate the policy circumstances of the respective period as well as possible.

**Fig. 2:**
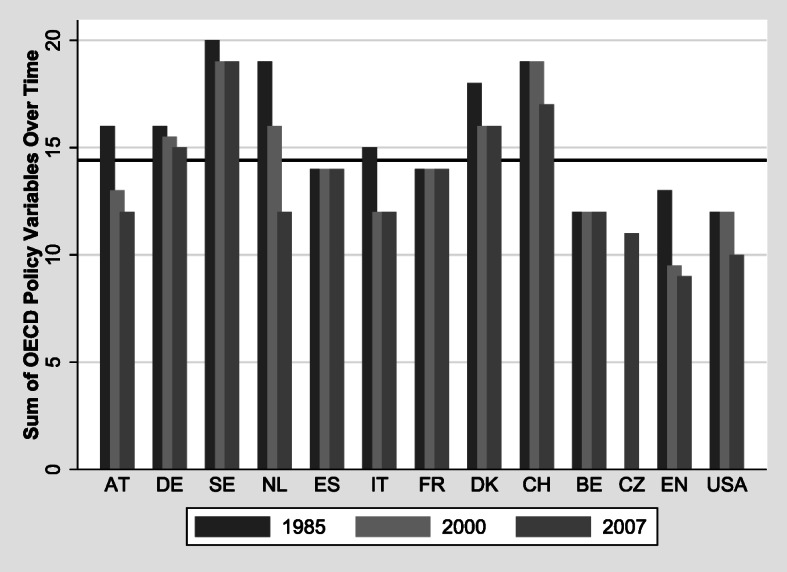
Generosity of DI systems over time and by countries: Austria (AT), Germany (DE), Sweden (SE), Netherlands (NL), Spain (ES), Italy (IT), France (FR), Denmark (DK), Switzerland (CH), Belgium (BE), Czech Republic (CZ), England (EN), and United States (USA). *Source:* Own calculation based on OECD (2003, 2010).

DI generosity, measured as the sum of these OECD policy indicators, varies substantially across countries (Fig. 2). Sweden, Denmark, and Switzerland reveal high OECD policy scores at all points in time, reflecting above-average generosity of their DI systems. In contrast, four countries remain below the average generosity level: Belgium, England, the United States, and the Czech Republic. Some countries started with an above-average level of generosity—for example, the Netherlands and Austria—but show below-average levels of DI benefit generosity today. DI generosity decreased between 1985 and 2007 in almost all countries, meaning that in general, the systems have become less generous, reflecting the incisive reforms mentioned in the Introduction. The exceptions are Spain, France, and Belgium, where the overall level of generosity has remained stable.

## Empirical Results: Within-Country Variation

### Regression Analysis

We estimate the equations presented earlier by multivariate probit regression analysis based on the pooled sample of all 13 countries. Pooling is possible because of the strict harmonization within SHARE and our additional harmonization among SHARE, ELSA, and HRS (see the Data section as well as section B of the online appendix). Regression results are displayed in Table 2. We compare some of them with the bivariate correlations in Table 1. Table 2 shows average marginal effects. Column 1 corresponds to Eq. (2) and includes subjective health. Columns 2a and 2b correspond to Eq. (3). They do not include subjective health and are the basis for predicting $\hat{WD}$ according to Eq. (4). As opposed to column 2a, column 2b includes the full set of life events, many of them only available in a subset of our data. Finally, we use the predictive values as regressors in columns 3a, 3b, and 3c, which correspond to Eq. (6). Columns 3b and 3c include the full set of life events, column 3c is an instrumental-variable variant of column 3b to be explained further later. Other regressors include demographic variables (**ENV**) and DI policy indicators (**POL**). Columns 2a and 3a show our baseline specifications.

**Table 2:** Determinants of WD and DI

	WD	WD	WD	DI	DI	DI
	(1)	(2a)	(2b)	(3a)	(3b)	(3c)
WD Predicted by (2a)				0.345**		0.367**
				(0.030)		(0.054)
WD Predicted by (2b)					0.350**	
					(0.041)	
Family and Social Environment (**ENV**)						
Age	–0.043**	–0.020	–0.100	0.032*	0.001	–0.046
	(0.015)	(0.019)	(0.069)	(0.016)	(0.065)	(0.065)
Age^2^ / 100	0.391**	0.193	0.865	–0.273	–0.029	0.375
	(0.127)	(0.161)	(0.577)	(0.141)	(0.559)	(0.555)
Female	–0.017*	–0.049**	–0.023	–0.011	–0.027	–0.017
	(0.009)	(0.010)	(0.015)	(0.007)	(0.014)	(0.015)
Education, high	–0.009	–0.052**	–0.026*	–0.030*	–0.022	–0.019
	(0.012)	(0.012)	(0.011)	(0.013)	(0.019)	(0.019)
Education, medium	0.007	–0.015	–0.012	–0.013	–0.012	–0.013
	(0.009)	(0.011)	(0.012)	(0.010)	(0.011)	(0.011)
Single	0.020*	0.018*	0.014	0.041**	0.044**	0.051**
	(0.008)	(0.008)	(0.019)	(0.006)	(0.014)	(0.015)
Divorced	0.031**	0.030**	0.028	0.030**	0.025	0.030
	(0.008)	(0.010)	(0.017)	(0.004)	(0.015)	(0.015)
Widowed	0.020	0.011	–0.057*	0.030*	0.048	0.019
	(0.013)	(0.013)	(0.029)	(0.013)	(0.027)	(0.027)
Health (*SUBJ*, **OBJH**)						
Self-reported health	0.111**					
	(0.015)					
ADL	0.068**	0.093**	0.106**			
	(0.012)	(0.013)	(0.013)			
IADL	0.025**	0.028*	0.043			
	(0.009)	(0.012)	(0.032)			
Grip strength	–0.001**	–0.002**	–0.001			
	(0.000)	(0.001)	(0.001)			
Grip strength missing	–0.047**	–0.102**	–0.057			
	(0.015)	(0.023)	(0.034)			
EURO-D	0.015**	0.036**	0.033**			
	(0.002)	(0.003)	(0.005)			
Recall abilities	–0.000	–0.003*	–0.003			
	(0.001)	(0.001)	(0.002)			
Life Course (**LIFE**)						
Childhood illnesses	0.019**	0.021**	0.018**			
	(0.004)	(0.005)	(0.004)			
Adulthood illnesses	0.039**	0.064**	0.069**			
	(0.002)	(0.008)	(0.010)			
Periods of poor health			0.055**			
			(0.007)			
Working gaps			0.101**		0.034	0.104**
			(0.032)		(0.027)	(0.023)
Low number of jobs			–0.017		–0.028*	–0.035**
			(0.013)		(0.012)	(0.012)
High number of jobs			0.014		–0.003	0.003
			(0.013)		(0.008)	(0.008)
Childhood number of rooms			0.001		–0.002	–0.002
			(0.004)		(0.003)	(0.003)
Childhood number of books			–0.003		–0.002	–0.002
			(0.005)		(0.005)	(0.005)
OECD Sum Score (**POL**)				0.010**	0.006*	0.006
				(0.004)	(0.003)	(0.004)
Pseudo-*R*^2^	.29	.22	.23	.19	.21	.18
*N*	29,571	29,571	4,697	29,571	4,697	4,699
Joint Significance Test Age and Age^2^ / 100^a^	11.42	2.73	3.42	5.04	0.94	1.20
*p*	.0033	.2557	.1806	.0803	.6247	.5475

*Notes:* The table presents marginal effects of probit estimations. Standard errors, clustered by country, are shown in parentheses. The table is based on HRS, ELSA, and SHARE including the following countries: Austria, Germany, Sweden, the Netherlands, Spain, Italy, France, Denmark, Switzerland, Belgium, the Czech Republic, England, and the United States.

^a^χ^2^ (*df* = 2)

*p < .05; **p < .01

Because WD may be the result of a long-lasting process, demographics and current health measures might not appropriately capture the effect on WD. We therefore include additional life course variables about early childhood conditions and the work history in columns 2b and 3b of Table 2. These variables are available for only SHARE and ELSA and only for respondents having participated in both Wave 3 and Wave 5/Wave 6 of SHARE/ELSA, respectively, which leads to a substantial reduction in our sample size to 4,703 observations.

The models of our baseline specifications in columns 2a and 3a of Table 2 explain 22% and 19% of the total variation for WD and DI receipt, respectively. WD as well as DI benefits receipt increase with age only if the bivariate correlation is considered (Table 1). However, this correlation disappears when health is taken into account (see the last row in Table 2 for a joint test of linear and squared age). Women are more likely to report a work limitation, but DI benefit receipt is almost equal among men and women in Table 1. Conditional on other variables, however, women are less likely to self-report a WD and also have a lower probability of receiving DI benefits (Table 2). This is in line with previous findings (OECD 2003) and can be explained by a lower labor market participation of women in general and the fact that many countries have lower eligibility ages for early retirement for women compared with men. Thus, for women, alternative routes to early retirement are available.

There is a clear education gradient for both variables in the bivariate statistics. Among those with low education, more persons report WD and receive DI (27.9% and 14.1%, respectively) than in the middle (24.3% and 10.9%, respectively) and high education group (16.1% and 6.1%, respectively). However, the gradient becomes much less pronounced when we control for differences in health (Table 2). The higher the education level, the smaller is the probability of reporting a WD or receiving DI benefits. This can be explained by the different occupational types. If disability benefits are granted also on the basis that a specific job can still be done, then those in low-skilled but physically demanding jobs are more likely to be granted benefits.

Marital status plays an important role for the receipt of DI benefits. In the group of married persons, only 8.0% receive DI. In the other marital status groups (singles, widowed, and divorced), approximately 16% are enrolled in DI benefits (Table 1). The pattern remains in the multivariate regressions. One explanation is that some secondary DI benefit programs are means-tested, and the income of the partner is taken into consideration (e.g., income-based employment and support allowance in England, Supplemental Security Income in the United States, and noncontributory disability pension in Spain). Married individuals are also less likely to report WD compared with single, divorced, and widowed persons. Here, the reasons could be related to help from spouses and healthier lifestyles among married individuals.

As expected, current health is strongly related to reporting WD and receiving DI pensions in the bivariate correlations reported in Table 1. The worse the health category is, the more persons are restricted and receive an income replacement. The share of persons with WD and receiving DI is especially high for low categories of self-reported health measures (self-reported health, ADL, IADL). A bad health status according to objective health measures also reveals a higher share of individuals with WD and more DI recipients (grip strength, recall abilities). Our benchmark regression (column 2a in Table 2) shows substantial and significant relations between WD and the objective health measures, such as grip strength and the EURO-D depression scale.^8^ In turn, the receipt of DI benefits is strongly influenced by $\hat{WD}$ and by the DI policy indicators (column 3a in Table 2).^9^ If the OECD score describing the generosity of the disability pension system increases by 1 point, on average, the probability of receiving a DI pension increases by roughly 1%.^10^

Current or very recent health measures, as broadly as they may be measured, may not appropriately capture the full impact of poor health on employability. Work disability may rather be the result of a long-lasting process of becoming sick and finally unable to work. We therefore include lifetime health indicators that describe childhood and adulthood health status in our regression. These variables are highly significant determinants of reported WD even after controlling for current health. Among those who report more than five childhood illnesses, 59% report WD, and 32% receive DI at older ages. Among those with more than five adulthood illnesses, 79% report WD, and 45% currently receive DI benefits.

Thus, health problems experienced over the life course and even as early as childhood are important drivers of later-life working capacity and the need to rely on DI benefits. This is an important result for two reasons. First, from a methodological point of view, health indicators measured as early as childhood are much less likely to be endogenous to labor market outcomes due to the time sequence of events. Thus, the measured effects can more convincingly be interpreted causally. Second, from a policy perspective, health interventions that target children when young do not only matter for their health at that point in time but have (positive) long-term impacts for health and labor market participation later in life.

Columns 2b and 3b of Table 2 take other life course features as part of **LIFE** into account. We include the number of gaps in the work history in which a person was sick or disabled. The results are positively significant and as expected. The more an individual experienced work gaps due to sickness during their career, the higher the probability of reporting WD and of receiving DI benefits later in life. We further include a binary variable indicating whether respondents experienced an extended period of poor health, which also has a positive and significant effect on both dependent variables. The number of jobs during the working life in general does not have a significant effect on WD. However, individuals with a particularly low number of jobs have a high likelihood of receiving DI benefits probably because they left the labor market early in their career.^11^ The socioeconomic status during childhood is measured by the number of books and the number of rooms per person in the accommodation. These indicators of early childhood socioeconomic circumstances are not related to WD or DI receipt. One explanation is that we already control for childhood health, which is directly related to the health and working life situation when old.

Column 3c is a variant of 3b that uses the predicted value of WD from column 2a without the life course variables rather than from 2b, which includes these variables. These additional exclusion restrictions should strengthen the identification of the second stage. Indeed, the coefficient of WD is slightly larger, but the standard error also increases, and the differences between 3b and 3c are small.

### Variance Decomposition

**Fig. 3:**
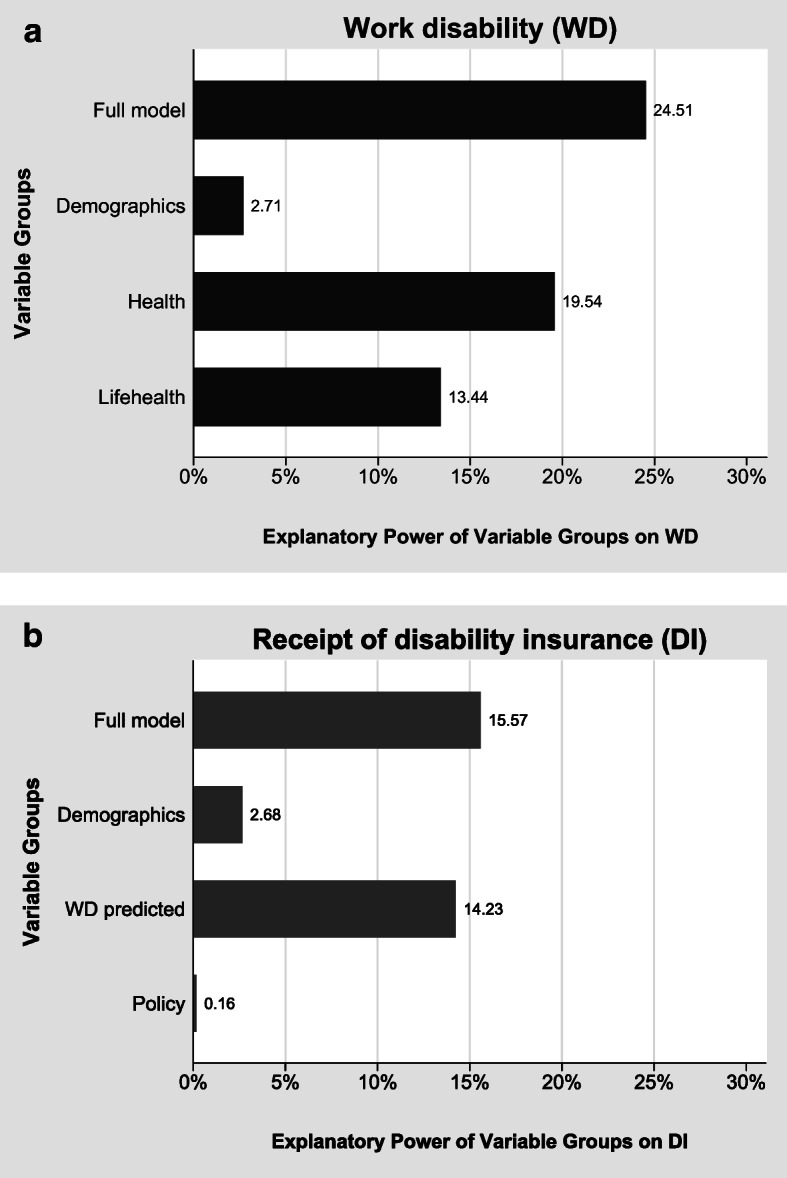
Variance decomposition for the probability of reporting WD and receiving DI benefits. *Source:* Own calculations based on weighted data from SHARE Wave 5, ELSA Wave 6, and HRS Wave 11. Based on linear regression models (*N* = 29,788).

To understand the contribution of different variable groups of explaining the variation in WD and DI receipt, we perform a variance decomposition analysis.^12^ Fig. 3 (upper panel) shows the variance decomposition of the individual variation in WD. The explanatory power of the full model is 25%. Most of the variation in WD (20%) can be explained by current health status. The second most important variable group consists of the life course health indicators. They can explain 13% of the total variation, indicating that health problems that occur early in life matter greatly for work disabilities later in life. Demographics (3%) have only small explanatory power for individual-level WD.

The lower panel of Fig. 3 shows how much of the variation in DI benefit receipt is explained by each variable group. The full model explains 16% of the variation in the data, which is less than in the case of self-assessed WD. However, the overall pattern is rather similar. The most important determinant of DI benefit receipt is the predicted WD: 14% of the variation is explained by $\hat{WD}$. Basic demographics account for only 3% of the variation. The policy indicators explain less than 1% of the individual variation in DI benefit receipt.

**Fig. 4:**
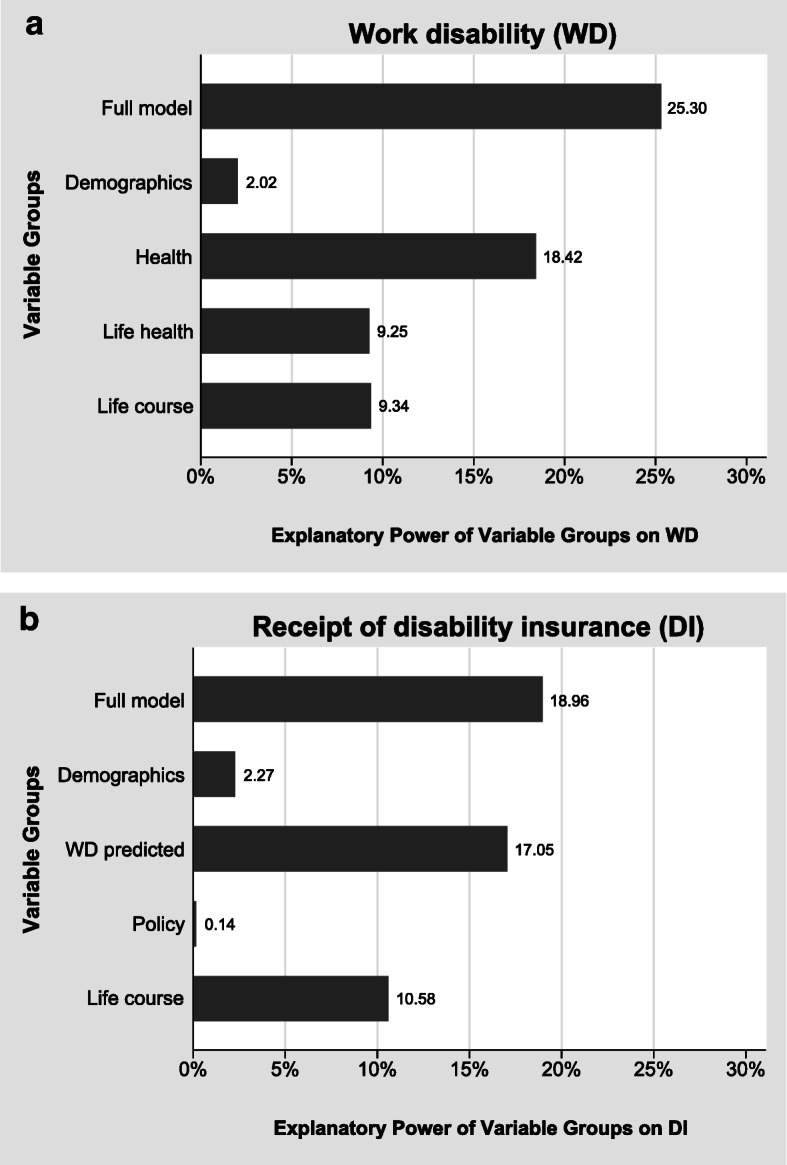
Variance decomposition for the probability of reporting WD and receiving DI benefits. *Source:* Own calculations based on weighted data from SHARE Waves 3 and 5, and ELSA Waves 3 and 6. Based on linear regression models (*N* = 4,703).

In Fig. 4, we present the results of the variance decomposition for the expanded models of columns (2b) and (3b) in Table 2. The full models including the additional life course indicators explain 25% (19%) of the total variance in case of WD (DI). As before, the variables measuring current health are the most important determinants of WD and DI benefit receipt. In the case of WD, life course health and other life course indicators are about equally important: both sets of variables explain about 9% of the total variance each. In the case of DI benefit receipt, the life course indicators even explain 11% of the total variance.

## Match Quality

This section assesses how well DI benefit receipt matches predicted work disability, $\hat{WD}$, in each country. This assessment is not straightforward because $\hat{WD}$ and DI receipt are measured on different scales (i.e., the thresholds in Eqs. (1) and (5) have different values). Hence, our data do not allow us to obtain an absolute comparison of the matching success across the countries in our sample. However, we can assess the relative match quality by normalizing the two underlying scales to have a common average value. This is achieved by the following procedure: we define

7 $$ {DI}_i=1\ \mathrm{if}\ \Phi \left({\mathbf{x}}_i\right)>0\ \mathrm{and}\ {\hat{WD}}_i=1\ \mathrm{if}\ \Phi \left({\mathbf{x}}_i\right)>\uptheta, $$

where *i* denotes the individual, **x** represents the regressors in Eqs. (3) and (6), and θ is set such that the population average of $\hat{WD}$is equal to the population average of *DI*. Because the threshold values are the same for each country, this procedure eliminates the overall difference between the scales of $\hat{WD}$ and DI but preserves country-specific differences in match quality relative to the overall scale.^13^

**Fig. 5:**
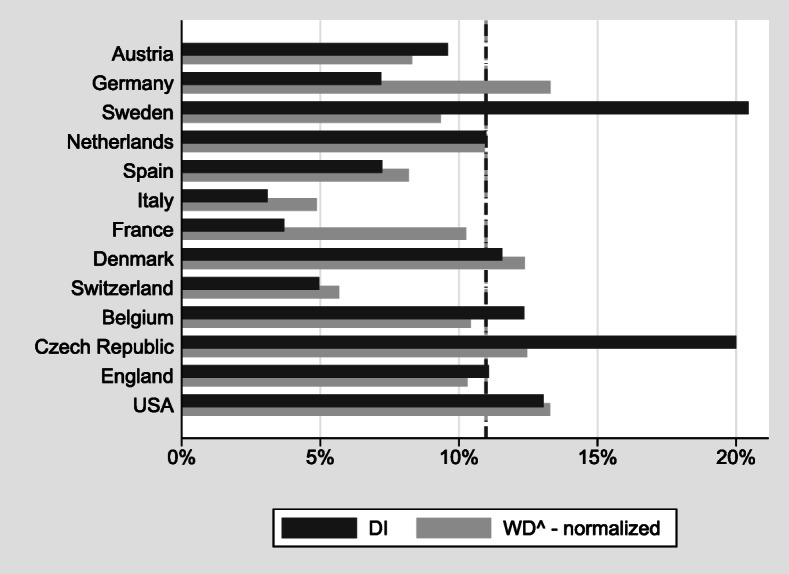
WD and DI receipt (normalized). *Source:* Own calculations based on weighted data from SHARE Wave 5, ELSA Wave 6, and HRS Wave 11.

Figure 5 shows that in many countries, the rates of self-reported WD and DI benefit receipt match each other quite well despite a couple of notable exceptions: Sweden and the Czech Republic appear very generous in granting DI benefits. Here, DI benefit rates are much higher than the rates of self-reported disability. The opposite is the case for France, Italy, Spain, and Germany, where the fraction of persons with self-reported disabilities is much higher than those receiving DI benefits.

**Fig. 6:**
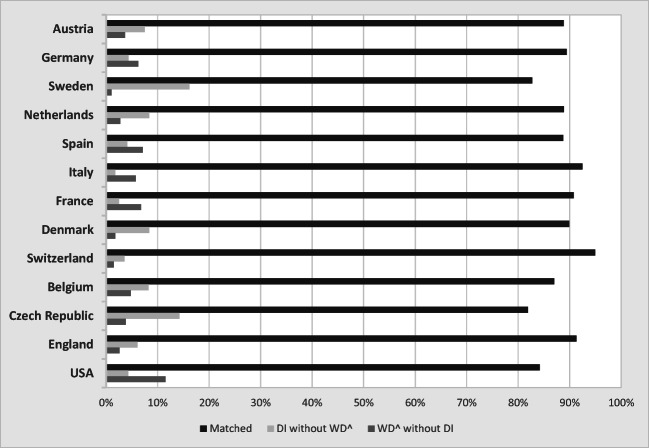
Match between predicted WD and DI receipt*. Source:* Own calculations based on weighted data from SHARE Wave 5, ELSA Wave 6, and HRS Wave 11.

**Fig. 7:**
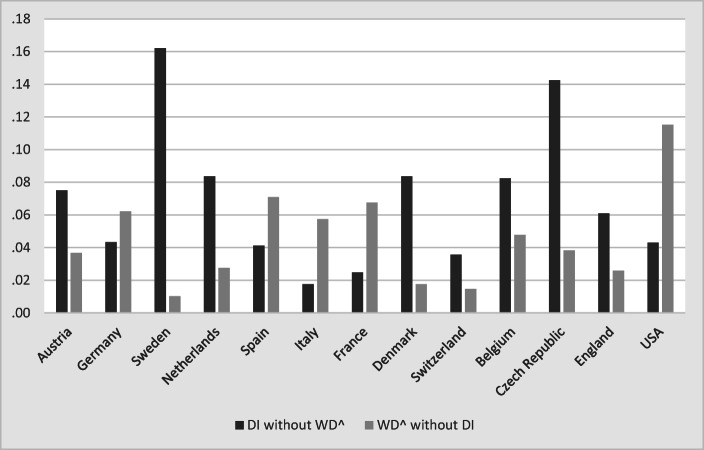
Frequency of mismatches by country. *Source:* Own calculations based on weighted data from SHARE Wave 5, ELSA Wave 6, and HRS Wave 11.

Figures 6 and 7 take a more precise look by basing the comparison between $\hat{WD}$ and *DI* on each individual. Across all 29,571 individuals in 13 countries, 88.5% are matched in the sense that they have a predicted WD and receive DI benefits or have no predicted WD and do not receive DI. However, at the same time, about 4.6% of individuals have a predicted WD but do not receive DI benefits. In turn, 6.9% receive DI benefits but do not have a predicted WD. Figure 6 shows that the frequency of a match is highest in Switzerland (95%) and lowest in the Czech Republic (82%).

Figure 7 provides a closer look at the mismatches. Italy, France, and the United States stand out with a high fraction of individuals who have a predicted WD and do not receive DI benefits. In the United States, it is almost 12% of the population. In contrast, Sweden and the Czech Republic give DI benefits to more than 16% and 12%, respectively, of all individuals aged 50–65, although these beneficiaries do not have limitations in their predicted ability to work.

## Counterfactual Simulations to Explain the Between-Country Variation in WD and DI

Finally, we would like to understand the large differences between the prevalence of WD and the receipt of DI benefits across countries. Previous work has shown that although health explains a great deal of the within-country variation in early retirement at any point in time, hardly any relationship exists between disability benefit receipt and average population health in a cross-national perspective (Börsch-Supan 2005). Moreover, the time series correlation between old-age labor force participation and objective measures of population health, such as mortality rates, is very low (Börsch-Supan and Jürges 2012).

We reproduce these results using the same regressions as reported in Table 2 to predict average DI and WD rates by country. For the baseline prediction, we use all variables as they are. For the counterfactual simulations, we set specific variable groups (demographics, health, policy indicators) to the average for all countries. Italy, for instance, has an older population than the European average, and Denmark has a younger population. In the upcoming counterfactual simulations, we remove these demographic differences. In this way, we predict which share of our sample would report a WD and take up DI benefits if everybody had the same characteristics as the average of all countries.

**Fig. 8:**
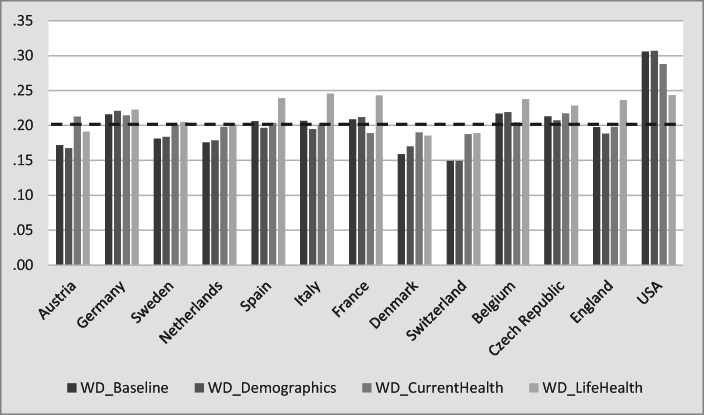
Counterfactual simulation for WD. *Source:* Own calculations based on weighted data from SHARE Wave 5, ELSA Wave 6, and HRS Wave 11.

Figure 8 compares the prevailing predicted WD rates with counterfactual simulation results if the demographic variables, current health, and lifetime health are set to the averages across all 13 countries. The average WD rate is about 20% and is indicated by the dashed line.^14^ Taking account of demographic differences between countries does not make a substantive difference. This is different for health, especially lifetime health. In the European countries with good average population health, WD rates would be higher when set to the average health status. However, the United States—with population health status that is worse than average—would reveal lower rates of WD when simulating a relatively better health status.

**Fig. 9:**
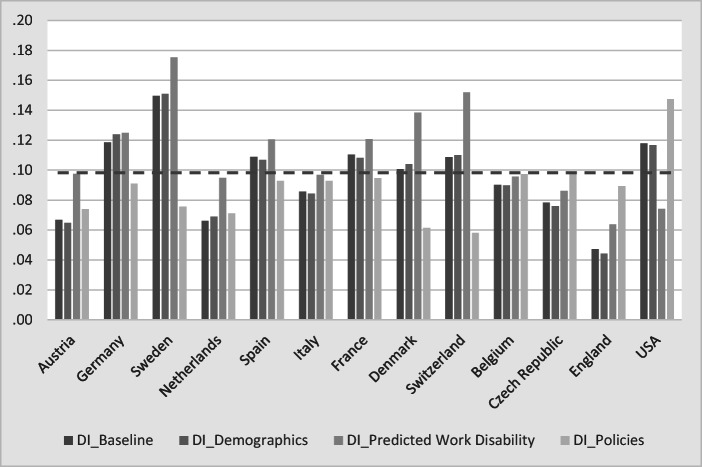
Counterfactual simulation for DI. *Source:* Own calculations based on weighted data from SHARE Wave 5, ELSA Wave 6, and HRS Wave 11.

Figure 9 displays the main result of this section and compares baseline and counterfactual simulation results of DI benefit receipt. The average DI rate is 9.6%; countries are sorted by their DI rate. Demographic differences between countries are relatively small, as indicated by the differences between the first and the second bar for each country. Hence, demographic differences can be ruled out as the main cause of the between-country variation in DI rates.

Equalizing predicted WD generates more changes in the variation of DI receipt than equalizing demographics. In countries with a good average population health—such as Sweden, Denmark, and Switzerland—DI enrollment rates would be much higher if they had the average WD status, and the converse would hold for the United States. If health were the main determinant for the variation of DI enrollment rates, the predicted counterfactual rates should move toward the average predicted DI rate (dashed line). However, as Fig. 9 shows, this is not the case. Hence, differences in health are not the explanation for the between-country variation of DI benefit receipt. More formally, the average deviation from the dashed line, measured as root mean square error, increases rather than decreases from the baseline value (2.6%) to the simulation, which counterfactually eliminates cross-national differences in WD (3.0%).

The opposite is the case if we set the indicator variables for the DI benefit generosity to the average across all 13 countries—that is, if we equalize DI institutions across countries. The root mean square error now declines to 2.1%.^15^ The pattern of DI uptake rates changes strikingly when equalizing the policy variables: the institutional environment in countries such as England, the Czech Republic, or the United States is counterfactually assumed to become more generous, but countries such as Sweden or Denmark become less generous when granting DI benefits. In most countries, the counterfactual simulation leads to DI enrollment rates that approach the overall average DI rate. This is especially visible in the comparison between England (smallest DI rate at baseline = 4.7%) and Sweden (highest DI rate = 15%). They are much closer to each other and the average (dashed line) when we counterfactually assume that these two countries have the same DI benefit generosity. Exceptions are the most generous and at the same time the healthiest countries (such as Switzerland and Denmark) where the simulated DI enrollment rates decrease far below the average DI rate of 9%. The contrary holds for the United States, which has one of the most restrictive DI regulations and on average an unhealthy population. In this case, applying the average degree of generosity would increase the incentives to enroll in DI benefits, and the simulated DI uptake rates grow up to almost 15%.

The strong effect of the policy variables in the counterfactual simulation exercise (Fig. 9) seems to contradict the small effect of the policy indicators in Figs. 3 and 4. This is not the case but reflects the different nature of the variation within and between countries. The results shown in Figs. 3 and 4 are dominated by within-country variation, whereas the counterfactual simulations exhibit only between-country variation. Within each country, DI policies have changed over time (Fig. 2), but this variation is small relative to the within-country variation of individual health. In contrast, all within-country variation is eliminated in Fig. 9, which rests purely on between-country variation. Between the countries in our sample, however, the variation of DI policies is much larger than the cross-national differences in health and sociodemographics.

## Conclusions

The objective of DI is to provide basic protection for those who suffer from work disabilities. This protection has two dimensions: protection from poverty by income support and protection from deteriorating health by permitting individuals to retire early. This study has evaluated both of the objectives of DI using harmonized data from SHARE, ELSA, and HRS, including life history variables. At the individual level within each of the 13 countries in this study, we find strong and equidirectional effects of current health and sociodemographic circumstances on reporting WD and receiving DI benefits.

Moreover, health experienced early in life matters a great deal for reported WD and DI receipt later in life. The life health variables are statistically highly significant and have large effect sizes. They are the second most important group of variables explaining WD and DI after current health indicators. Thus, health problems experienced over the life course are important drivers of later-life working capacity and the need to rely on DI benefits. Even illnesses experienced in childhood have long-term consequences. Social expenditures on health of children are therefore well spent given that they not only improve health but also have very long-term benefits for the onset of work disabilities and ultimately the reliance on DI benefit receipt.

Already on an individual level, we find that DI institutions matter significantly for DI receipt. This effect is identified by the variation over time captured in the life histories. When DI systems became less generous, the likelihood of receiving DI pensions decreased, holding health and sociodemographic indicators constant. On the individual level, this effect is small compared with the variables measuring individual health, as our variance decompositions show.

At the country level, however, the picture is dominated by factors describing the generosity of the DI systems. Country differences in demographic characteristics, such as population aging and health differences, contribute little in explaining the international variation in DI benefit receipt. In our counterfactual simulation exercises, DI enrollment rates approach the average DI rate when the policy variables are equalized. Exceptions are the healthiest and most generous countries, such as Switzerland and Denmark, on the one hand and the least healthy and most restrictive country, the United States, on the other hand.

The large country differences may not be due to DI policies alone. More work is necessary to understand the precise interactions and causal chains among labor market environment, DI policies, and long-term health effects, as well as the interactions between job characteristics and the medical and occupational assessment rules.

Given the large differences in the generosity and the prevalence of DI, and given the large costs of DI, the obvious next question is then whether the added expenses are well spent. Does a generous DI system improve individuals’ well-being and health? Will this permit reintegration into the labor market? Further research is also needed to better understand which countries are successful by providing special employment programs or flexible work schemes following up on DI benefit receipt.

## Electronic supplementary material

ESM (PDF 447 kb)

## Data Availability

All three data sets—SHARE, ELSA, and HRS—are available to all scientific users free of charge via their respective websites. The harmonization procedure used in this paper is documented in an extensive technical appendix available at https://www.mpisoc.mpg.de/en/social-policy-mea/publications/detail/publication/early-determinants-of-work-disability-in-an-international-perspective-2/.

## Footnotes

1 Details are explained later in the article.

2 Life histories are highly structured computer-assisted interviews that collect retrospective data on the most salient health, family, social, work, accommodation, and economic events from childhood to current age (Belli 1998). For more details, see section B of the online appendix.

3 For the definition of the statutory retirement ages, we gather information on the national pension systems. We create a binary variable indicating whether someone is above or below the national statutory retirement age, taking into account transitional arrangements of pension reforms separately for men and women (see Table A2, online appendix).

4 Table 1 provides descriptive statistics. Depending on the explanatory variables used, the analytic samples may slightly vary from the sample used for this table.

5 In ELSA and HRS, another depression index—the CES-D score—is used. SHARE contains the information needed for both the EURO-D and the CES-D score in Wave 1. Based on this information, we build a prediction rule for EURO-D by means of a linear regression and apply this rule to the HRS and ELSA data to obtain the predicted EURO-D scores.

6 Because information on marital status is given only if something changed since the last interview, we merge information from all previous waves. The same applies to educational level.

7 Using the five indicators separately does not substantially affect the results.

8 We are grateful for a referee pointing out that the mental state described by EURO-D may influence the subjective health assessment and thus can be correlated with the error term. We therefore run all regressions in Table 2 without EURO-D. Results remain robust in terms of statistical significance and economic substance.

9 Clustered standard errors account for the fact that this variable varies across countries only.

10 As a robustness check, we run a probit regression with country fixed effects instead of the OECD variables. As expected, the results for the other variable groups remain stable in size and sign. Results are reported in Table A5 in the online appendix. We also run a regression where we control for the five individual OECD indicators describing the DI pension systems. Results are reported in Table A6 in the online appendix.

11 This may create an endogeneity problem due to the correlation with DI benefit receipt. We also use number of jobs per total lifetime spent in employment, and results do not change.

12 The decomposition is based on linear regression models presented in Table A4 in the online appendix. The linear specification gives very similar results as the probit model presented earlier.

13 This assumption of a common θ is, of course, a strong one (cf. Kapteyn et al. 2007; Sen 2002).

14 The lower value compared with Fig. 1 is due to the different analytic sample.

15 We also conduct the same exercise using the five subscales of the OECD policy indicator, and the results are qualitatively the same.
