# Supplementary material for: Early Determinants of Work Disability in an International Perspective
Source: Demography. 2020 Aug 25;57(5):1853–79. doi: 10.1007/s13524-020-00902-7 (PMC7584547; doi:10.1007/s13524-020-00902-7)
Supplement: Supplementary file 1 — ESM (PDF 447 kb) [file 13524_2020_902_MOESM1_ESM.pdf]

MAX PLANCK INSTITUTE FOR
SOCIAL LAW AND SOCIAL POLICY
mea Munich Center for the Economics of Aging

Early determinants of work disability in an international perspective

ONLINE APPENDIX

A. Additional Tables

B. Technical Appendix – Harmonization process

Axel Börsch-Supan[abc], Tabea Bucher-Koenen[ade] and Felizia Hanemann[ab]

a: Munich Center for the Economics of Aging (MEA) at the Max Planck Institute for Social Law and Social Policy, Munich, Germany.

b: Department of Economics and Business, Technical University of Munich (TUM), Munich, Germany.

c: National Bureau of Economic Research (NBER), Cambridge, Mass.

d: ZEW – Leibniz Center for European Economic Research, Mannheim, Germany.

e: University of Mannheim, Germany.

A. Additional tables

Table A1: DI system indicators per country

1985	AT	BE	DK	FR	DE	IT	NL	ES	SE	CH	CZ	UK	USA
Benefit_system_coverage	5	3	5	3	2	3	4	1	5	5	n.a.	3	3
Minimum_disability_benefit	5	2	3	2	3	2	5	4	4	3	n.a.	1	1
Disability_benefit_generosity	1	1	4	3	2	3	5	4	5	4	n.a.	1	3
Medical_assessment_rules	3	2	4	2	4	2	1	0	4	5	n.a.	3	4
Vocational_assessment_rules	2	4	2	4	5	5	4	5	2	2	n.a.	5	1
SUM	16	12	18	14	16	15	19	14	20	19	n.a.	13	12

OECD (2003)

2000	AT	BE	DK	FR	DE	IT	NL	ES	SE	CH	CZ	UK	USA
Benefit_system_coverage	2	3	5	3	2	3	4	3	5	5	n.a.	3	3
Minimum_disability_benefit	3	2	3	2	5	2	5	4	5	4	n.a.	1	1
Disability_benefit_generosity	2	1	4	3	2	3	5	4	5	4	n.a.	1	3
Medical_assessment_rules	1	2	3	2	3	1	1	0	3	4	n.a.	3	4
Vocational_assessment_rules	5	4	1	4	3,5	3	1	3	1	2	n.a.	1,5	1
SUM	13	12	16	14	15,5	12	16	14	19	19	n.a.	9,5	12

OECD (2003)

2007	AT	BE	DK	FR	DE	IT	NL	ES	SE	CH	CZ	UK	USA
Benefit_system_coverage	2	3	5	3	3	3	4	3	5	5	1	3	3
Minimum_disability_benefit	3	2	2	2	5	2	4	4	5	4	4	1	0
Disability_benefit_generosity	2	1	3	3	2	3	3	4	5	3	3	1	3
Medical_assessment_rules	1	2	4	2	3	1	1	0	3	3	2	3	4
Vocational_assessment_rules	4	4	2	4	2	3	0	3	1	2	1	1	0
SUM	12	12	16	14	15	12	12	14	19	17	11	9	10

OECD (2010)

Table A2: Definition of statutory retirement ages per country

	Women	Men
Austria	60	65
Belgium	60 if year of birth <1936<br>61 if year of birth >=1936 & <1938<br>62 if year of birth >=1938 & <1940<br>63 if year of birth >=1940 & <1942<br>64 if year of birth >=1942 & <1944<br>65 if year of birth >=1942 & <1944<br>65 if year of birth >=1944	65
Czech Republic	57 if year of birth <1941<br>58 if year of birth >=1941 & <1944<br>59 if year of birth >=1944 & <1947<br>60 if year of birth >=1947 & <1950<br>61 if year of birth >=1950 & <1953<br>62 if year of birth >=1953 & <1956<br>63 if year of birth >=1956	60 if year of birth <1941<br>61 if year of birth >=1941 & <1947<br>62 if year of birth >=1947 & <1953<br>63 if year of birth >=1953 & <1959<br>64 if year of birth >=1959 & <1965<br>65 if year of birth >=1965 & <1971<br>66 if year of birth >=1971 & <1977<br>67 if year of birth >=1977
Denmark	65<br>67 if year of birth <=1939	65<br>67 if year of birth <=1939
France	65 if year of birth <=1919<br>60 if year of birth >=1951	65 if year of birth <=1919<br>60 if year of birth >=1951
Germany	65 if year of birth<1958	65 if year of birth<1958
Italy	55 if year of birth <1939<br>56 if year of birth =1939<br>57 if year of birth =1939<br>58 if year of birth =1940<br>59 if year of birth =1940<br>60 if year of birth >=1941	60 if year of birth <1934<br>61 if year of birth =1934<br>62 if year of birth =1934<br>63 if year of birth =1935<br>64 if year of birth =1935<br>65 if year of birth >=1936
Netherlands	65	65
Spain	65	65
Sweden	65	65
Switzerland	62<br>63 if year of birth >=1956	65
United Kingdom	60 if year of birth<1951<br>61 if year of birth<1952	65
United States	65 if year of birth <=1937<br>66 if year of birth >=1937 & <1943<br>67 if year of birth >=1943	65 if year of birth <=1937<br>66 if year of birth >=1937 & <1943<br>67 if year of birth >=1943

Source: Own elaboration

Table A3: Definition of Disability Benefits

Austria	Staatliche Invaliditäts- bzw. Berufsunfähigkeitspension, Versehrtenrente oder Krankengeld (aus der Haupt- und Nebenbeschäftigung)
Belgium	Wettelijke/ Aanvullende uitkering bij ziekte of invaliditeit of wettelijke uitkering bij beroepsziekte of arbeidsongeval; Une allocation/pension maladie/invalidité/incapacité légale, Une deuxième assurance maladie/invalidité/incapacité légale
Czech Republic	Státní invalidní důchod, nemocenské dávky
Switzerland	Rente de l''assurance invalidité (AI); Rente der Invalidenversicherung (IV); Rendita invalidità AI
Germany	Erwerbsminderungsrente bzw. Beamtenpension wegen Dienstunfähigkeit, oder Krankengeld
Denmark	Førtidspension, herunder sygedagpenge
Spain	Pensión pública de invalidez/incapacidad o prestación pública por enfermedad, Segunda pensión pública de invalidez/incapacidad o segunda prestación pública por enfermedad; Pensió pública d''invalidesa / incapacitat o prestació pública per malaltia, Segona pensió pública d''invalidesa / incapacitat o segona prestació pública per malaltia
France	Une pension d'invalidité publique (y c. rente d'accident du travail et allocation supplémentaire d'invalidité)
Italy	Indennità pubblica di disabilità; pensione di invalidità, incapacità (incluso assegno di accompagnamento)
Netherlands	WAO, Waz, WIA, of ander invaliditeitspensioen
Sweden	Sjukersättning (förtidspension) eller sjukpenning
England	Incapacity benefits (previously invalidity benefits), Employment and Support Allowance, Severe Disablement Allowance SDA, Statutory sick pay SSP, Attendance Allowance, Disability Living Allowance, Industrial Injuries Disablement benefits
United States	SSDI and SSI disability pension

Table A4: Determinants of WD and DI - linear specification

		WD	WD	WD	DI	DI
		(1)	(3a)	(3b)	(2a)	(2b)
Demographics	Age	-0.051	-0.029	-0.091	0.033	0.003
		(0.015)**	(0.019)	(0.076)	(0.018)	(0.064)
	Age²/100	0.452	0.270	0.796	-0.289	-0.054
		(0.132)**	(0.170)	(0.638)	(0.156)	(0.550)
	Female	-0.026	-0.056	-0.028	-0.011	-0.030
		(0.010)*	(0.010)**	(0.015)	(0.008)	(0.015)
	Education_high	-0.009	-0.054	-0.028	-0.016	-0.011
		(0.013)	(0.013)**	(0.010)*	(0.014)	(0.018)
	Education_medium	0.004	-0.018	-0.013	-0.014	-0.012
		(0.010)	(0.012)	(0.013)	(0.013)	(0.013)
	Single	0.022	0.022	0.014	0.044	0.059
		(0.007)*	(0.008)*	(0.021)	(0.008)**	(0.020)*
	Divorced	0.037	0.035	0.036	0.030	0.024
		(0.010)**	(0.011)**	(0.021)	(0.006)**	(0.021)
	Widowed	0.019	0.010	-0.060	0.033	0.061
		(0.015)	(0.014)	(0.025)*	(0.017)	(0.032)
Health	Self-reported health	0.116				
		(0.012)**				
	ADL	0.089	0.108	0.118		
		(0.007)**	(0.007)**	(0.008)**		
	IADL	0.036	0.038	0.044		
		(0.008)**	(0.010)**	(0.018)*		
	Grip strength	-0.001	-0.002	-0.002		
		(0.000)**	(0.001)**	(0.001)		
	Grip strength missing	-0.062	-0.118	-0.071		
		(0.017)**	(0.023)**	(0.032)*		
	EURO-D	0.022	0.044	0.040		
		(0.002)**	(0.004)**	(0.007)**		
	Recall abilities	-0.000	-0.003	-0.004		
		(0.001)	(0.001)*	(0.002)		
Life health	Childhood illnesses	0.022	0.023	0.018		
		(0.004)**	(0.005)**	(0.005)**		
	Adulthood illnesses	0.049	0.076	0.085		
		(0.004)**	(0.010)**	(0.011)**		
	Periods of poor health			0.071		
				(0.008)**		
	WD^				0.556	0.589
					(0.036)**	(0.068)**
Life course	Working gaps			0.139		0.073
				(0.038)**		(0.065)
	Low nr. of jobs			-0.016		-0.024
				(0.013)		(0.010)*
	High nr. of jobs			0.014		-0.007
				(0.013)		(0.010)
	Childhood nr. rooms			0.002		-0.003
				(0.004)		(0.003)
	Childhood nr. books			-0.001		-0.000
				(0.006)		(0.005)
	OECD sum score				0.012	0.006
					(0.005)*	(0.004)
	Constant	1.226	0.965	2.736	-1.110	-0.076
		(0.429)*	(0.536)	(2.234)	(0.501)*	(1.866)
	N	29,571	29,571	4,697	29,571	4,697

* *p*<0.05; ** *p*<0.01, Based on linear regression specification. Standard errors in parentheses, clustered standard errors by country. Based on HRS, ELSA and SHARE including the following countries: AT, DE, SE, NL, ES, IT, FR, DK, CH, BE, CZ, UK, USA, Reference categories: Male, low education, married.

Table A5: Probit specification with country-fixed effects

	DI
Age	0.024
	(0.010)*
Age²	-0.204
	(0.090)*
Female	-0.010
	(0.007)
Education_high	-0.036
	(0.009)**
Education_medium	-0.012
	(0.004)**
Single	0.041
	(0.007)**
Divorced	0.026
	(0.005)**
Widowed	0.031
	(0.012)*
WD^	0.352
	(0.017)**
AT	0.051
	(0.004)**
DE	0.009
	(0.002)**
SE	0.126
	(0.002)**
NL	0.064
	(0.003)**
ES	0.001
	(0.004)
IT	-0.070
	(0.004)**
FR	-0.042
	(0.004)**
DK	0.077
	(0.003)**
CH	0.008
	(0.004)
BE	0.056
	(0.003)**
CZ	0.102
	(0.004)**
UK	0.038
	(0.004)**
Pseudo R2	0.21
N	29,571

* p<0.05; ** p<0.01, Marginal effects of probit specification. Standard errors in parentheses, clustered standard errors by country. Based on HRS, ELSA and SHARE, including the following countries: AT, DE, SE, NL, ES, IT, FR, DK, CH, BE, CZ, UK, USA (reference category).

Table A6: Probit specification with five single OECD indicators

	DI
Age	0.031
	(0.015)*
Age²	-0.262
	(0.133)*
Female	-0.010
	(0.007)
Education_high	-0.036
	(0.011)**
Education_medium	-0.020
	(0.006)**
Single	0.042
	(0.007)**
Divorced	0.031
	(0.004)**
Widowed	0.029
	(0.013)*
Self-reported health	0.343
	(0.040)**
OECD_coverage	-0.005
	(0.014)
OECD_minimum	0.018
	(0.012)
OECD_di_generosity	0.012
	(0.016)
OECD_medical	0.022
	(0.015)
OECD_vocational	0.011
	(0.012)
Pseudo R2	0.19
N	29,571

* p<0.05; ** p<0.01; marginal effects of probit specification. Standard errors in parentheses, clustered standard errors by country. Based on HRS, ELSA and SHARE including the following countries: AT, DE, SE, NL, ES, IT, FR, DK, CH, BE, CZ, UK, USA.

B. Technical Appendix – Harmonization process

SHARE is a pan-European data set designed to analyze the process of population aging using cross-national comparisons within Europe and between Europe, America and Asia (Börsch-Supan et al. 2013). The first wave in 2004 included eleven European countries and more than 22,000 individuals aged 50 and older. In the subsequent waves, which are conducted biennially, more countries joined the project so that SHARE currently includes 20 European countries, covering the area from Sweden to Greece and Portugal to Estonia.

SHARE is modeled closely after the US Health and Retirement Study (see Juster & Suzman 1995), which was the first survey of this kind, and the English Longitudinal Study of Ageing (see Marmot et al. 2003) which followed the lead by HRS. The first wave of HRS was initiated in 1992 and the subsequent waves were conducted in a biennial course. The initial sample included 12,652 individuals living in the United States aged between 51 and 61 years and their spouses or partners. Since this sample ages with the time of the survey, new individuals were sampled as a refreshment sample in later waves in order to represent the younger age group. Until today, 11 waves of HRS data are available.

On the basis of the HRS survey, a longitudinal old age survey was implemented in England in 2002. The baseline sample contains 12,099 persons representing the population aged 50 and older in the United Kingdom (UK). Further refreshment samples were added in subsequent waves. Until now, 6 waves of ELSA data are available.

All three datasets are multidisciplinary household panel surveys including detailed information on health, socioeconomic status, work history and social networks. Researchers from HRS and ELSA have been participating in the design process of SHARE at all stages. About two-thirds of the variables in SHARE are identical to variables in ELSA and HRS, and most of the remainder is closely comparable. The harmonization of these variables in HRS, ELSA and SHARE enables us to conduct comparative analyses for different regions in Europe, the UK and the US.

We will use internationally comparable life-course data on health and socio-economic circumstances. The main work was to construct a database of retrospective life histories collected by SHARE and ELSA, and comparable early childhood and life-course data collected by HRS. Life histories are highly structured computer-assisted interviews which collect retrospective data on the most salient health, family, social, work, accommodation, and economic events from childhood to current age (Belli 1998), including markers for genetic predisposition such as parents' health conditions and life spans. They can be interpreted as a

short-cut to a life-long cohort study. While retrospective data have some limitations, the value of information obtained from life histories has nevertheless been proven to be great: validation studies have shown that recall data contain very valuable information even if people do not reproduce events from the past perfectly (Rubin 1996, Jürges 2005). In wave 3, the SHARE panel data has been enriched with detailed accounts of the respondents' life histories (SHARELIFE). By integrating this retrospective view, the living conditions in the preceding decades become accessible, thus granting various insights going back as far as into childhood. The SHARE life histories have been modeled in close cooperation with the ELSA life histories. We enrich the data by variables from SHARELIFE and ELSALIFE, especially on socioeconomic status in childhood, on illnesses during childhood and adulthood and on the employment history of the respondents. HRS does not feature such structured life histories yet but the normal questionnaire covers some retrospective variables describing early childhood conditions and salient events in adult life which permit cross-walking between SHARE, ELSA and HRS.

It is a time-consuming task to construct the corresponding variables based on different survey questions. Ex-ante harmonization with the questionnaire of HRS is an important prerequisite of ELSA and SHARE and great efforts have been made to deliver truly comparable data. However, country-specific deviations in wording, categories or the non-applicability of questions and modules are unavoidable. Therefore the comparability of items has to be checked thoroughly one by one. All variables taken from HRS, SHARE and ELSA are harmonized carefully. A detailed description of the harmonization process as well as a list of all variables and how they were combined can be found in the Technical Appendix of this paper (Table B. 1 - Table B. 4).

Several steps are implemented to harmonize one specific variable. First some characteristics of the required variable are examined in SHARE. We consider the corresponding question to that variable as well as the possible answers and therefore characteristics of the variable - in sense of dichotomy, categorization, values and so forth. Those characteristics are used to compare the corresponding variables included in the HRS and ELSA datasets. After the first step we search for an appropriate variable. For the HRS dataset the RAND file and documentation is reviewed. If we cannot find a variable that can be harmonized, we examine the codebook, which is accessible on the official HRS homepage. If a required variable is not included in the RAND dataset of HRS, but can be found in the codebook, we take the needed data from the core dataset. There is one core dataset for each wave of HRS. The procedure with the ELSA data is similar. We check the existing datasets for each wave and the documentation. After searching for an appropriate variable for harmonization, we compare the variable's characteristics in

SHARE, ELSA and HRS. If there are differences, for example in the values, the variables of HRS and ELSA are adjusted to the corresponding variable in SHARE. An easy example would be the coding of the gender variable (male=0 female=1 instead of male=1 female=2). Only if both questioning and the characteristics of the variable are comparable between the studies, it can be harmonized.

As base dataset we perform this procedure for the wave 5 of SHARE, wave 6 of ELSA and Wave 11 of HRS. We further include information from the life history interviews (Wave 3 in SHARE and Wave 3 in ELSA) and adapt available retrospective information from HRS. Some variables also need to be merged from former waves (e.g. years of education is not asked repeatedly or marital status but only if it changed between waves). After creating one harmonized dataset for each study in long format, all three datasets are appended so we have a harmonized dataset containing all three studies.

Table B1: Overview of variable groups used in regression analyses

Group	Variable	Description	Range	Categories	Available in SHARE	Available in ELSA	Available in HRS
Demographics	age	Age at time of interview	20-89	20-89	yes	yes	yes
	female	Gender	0-1	0. Male<br>1. Female	yes	yes	yes
	education_low	Education category	0-1	0. Not in low education category<br>1. In low education category (ISCED 0-2)	yes	yes	yes
	education_medium	Education category	0-1	0. Not in medium education category<br>1. In medium education category (ISCED 3-4)	yes	yes	yes
	education_high	Education category	0-1	0. Not in high education category<br>1. In high education category (ISCED 5-6)	yes	yes	yes
	single	Currently not married, divorced or widowed	0-1	0. Not single<br>1. Single	yes	yes	yes
	married	Currently married	0-1	0. Not married<br>1. Married	yes	yes	yes
	divorced	Currently divorced	0-1	0. Not divorced<br>1. Divorced	yes	yes	yes
	widowed	Currently widowed	0-1	0. Not widowed<br>1. Widowed	yes	yes	yes
Health	sphus	Self-reported health	1-5	1. Excellent<br>2. Very good<br>3. Good<br>4. Fair<br>5. Poor	yes	yes	yes
	iadl	IADL: number of limitations with instrumental activities of daily living	0-6	Difficulties with:<br>Using a map, preparing a hot meal, shopping for groceries, making telephone calls, taking medications and managing money	yes	yes	yes
	adl	ADL: number of limitations with activities of daily living	0-6	Difficulties with:<br>Dressing, eating, using the toilet, bathing and showering, getting in and out of bed, walking across a room	yes	yes	yes
	recall	Ten words list learning – sum first and delayed recall	0-10	0-10	Yes	yes	yes
	grip strength	Maximal Grip Strength (Kg)	0.5 - 90	0.5 – 90	yes	yes	yes
	grip strength missing	Flag variable if missing value was imputed	0-1	0. No value was imputed<br>1. Missing value was replaced by zero	yes	yes	yes
	eurod	Depression scale	0-11	0-11	yes	from cesd	from cesd

	Work disability (lim_work)	Health problem that limits paid work	0-1	0. No<br>1. Yes	yes	yes	yes
Life health	illnesses_ch	Childhood Illnesses	0-9	0-9	yes	yes	Yes
	illnesses_adult	Adulthood Illnesses	0-9	0-9	yes	yes	yes
Lifecourse others	working_gaps	Working gaps due to sickness	0-2	0-2	yes	yes	no
	poor_health	Number of period of very poor health	0-5	0. None<br>1. One<br>2. Two<br>3. Three<br>4. More than three<br>5. Have been ill or with disabilities for all or most of my life	yes	yes	no
	rooms_ch	Number of rooms when ten years old	0-50	0-50	yes	yes	No
	books_ch	Number of books when ten years old	1-5	1. None or very few (0-10 books)<br>2. Enough to fill one shelf (11-25 books)<br>3. Enough to fill one bookcase (26-100 books)<br>4. Enough to fill two bookcases (101-200 books)<br>5. Enough to fill two or more bookcases (more than 200 books)	yes	yes	No
	low_n_jobs	Number of jobs over lifetime	0-1	0. Not having had a low number of jobs<br>1. Having had a low number of jobs (1-2)	yes	yes	yes
	medium_n_jobs	Number of jobs over lifetime	0-1	0. Not having had a medium number of jobs<br>1. Having had a medium number of jobs (3-4)	yes	yes	yes
	high_n_jobs	Number of jobs over lifetime	0-1	0. Not having had a high number of jobs<br>1. Having had a high number of jobs (>5)	yes	yes	yes
Policy	oecd_coverage	Benefit system coverage	0-5	0. Employees<br>1. Labour force<br>2. Labour force with voluntary self-insurance<br>3. Labour force plus means-tested non-contr. scheme<br>4. Some of those out of the labour force (e.g. congenital)<br>5. Total population (residents)	Not for Estonia, Israel, Slovenia	yes	Yes
	oecd_minimum	Minimum disability benefit	0-5	0. 86-100%<br>1. 71-85%<br>2. 56-70%<br>3. 41-55%<br>4. 26-40%<br>5. 0-25%	Not for Estonia, Israel, Slovenia	yes	Yes
	oecd_di_generosity	Disability benefit generosity	0-5	0. RR < 50%, minimum not specified<br>1. RR < 50%, reasonable minimum<br>2. 75 > RR > = 50%, minimum not specified<br>3. 75 > RR > = 50%, reasonable minimum	Not for Estonia, Israel, Slovenia	yes	Yes

				4. RR > = 75%, minimum not specified<br>5. RR > = 75%, reasonable minimum			
	oecd_medical	Medical assessment rules	0-5	0. Insurance team and two-step procedure<br>1. Team of experts in the insurance<br>2. Insurance doctor exclusively<br>3. Insurance doctor predominantly<br>4. Treating doctor predominantly<br>5. Treating doctor exclusively	Not for Estonia, Israel, Slovenia	yes	Yes
	oecd_vocational	Vocational assessment rules	0-5	0. All jobs available taken into account, strictly applied<br>1. All jobs available taken into account, leniently applied<br>2. Current labour market conditions are taken into account<br>3. Own-occupation assessment for partial benefits<br>4. Reference is made to one's previous earnings<br>5. Strict own or usual occupation assessment	Not for Estonia, Israel, Slovenia	yes	Yes
	oecd_sum	Sum of five OECD indicators	9-20	9-20	Not for Estonia, Israel, Slovenia	yes	Yes

Table B2: Detailed list of harmonized variables

Variable	Description	SHARE	ELSA	HRS
Disability benefits				
dis1	disability benefits	X	x	x
dis1_year	first year received disability benefits	X		x
Identifiers (merging…)				
mergeid	Identifier in SHARE	X		
idauniq	Identifier in ELSA		x	
hhidpn	Identifier in HRS			x
study	study identifier	X	x	x
Demographic				
country	Country identifier	X	x	x
yrbirth	Year of birth	X	x	x
age	age (max. 90)	X	x	x
gender	Gender	X	x	x
married	Is respondent married?	X	x	x
ever_married	Has respondent ever been married?	X	x	x
divorced	Is respondent divorced?	X	x	x
ever_divorced	Has respondent ever been divorced?	X	x	x
widowed	Is respondent widowed?	X	x	x
ever_widowed	Has respondent ever been widowed?	X	x	x
Education				
dn041_	years of education	X	x	x
educat	education category	X	x	x
Job				
numberjobs	number of jobs	X	x	x
working_gaps	number of working gaps	X	x	x
ep027_	My job is physically demanding.	X	x	x
ep028_	I am under constant time pressure due to a heavy workload.	X	x	x
ep029_	I have very little freedom to decide how I do my work.	X	x	
ep030_	I have an opportunity to develop new skills.	X	x	x
ep031_	I receive adequate support in difficult situations.	X	x	x
ep032_	I receive the recognition I deserve for my work.	X	x	x
ep033_	Considering all my efforts and achievements, my salary is/earnings are adequate	X	x	x
ep034_	Poor prospects for (main) job advancement	X	x	x
ep035_	Poor (main) job security	X	x	x
lowcontrol_ci	=1 low control (separately calculated for each country)	X	x	x
ERI	Effort-reward imbalance (>1 poor quality of work)	X	x	x
ERIi	=1 poor quality of work	X	x	x
ERIci	=1 poor quality of work (separately calculated for each country)	X	x	x
ep027_main	SHARE main job: My job is physically demanding.	X		
ep028_main	SHARE main job: I am under constant time pressure due to a heavy workload.	X		
ep029_main	SHARE main job: I have very little freedom to decide how I do my work.	X		
ep030_main	SHARE main job: I have an opportunity to develop new skills.	X		

ep031_main	SHARE main job: I receive adequate support in difficult situations.	X		
ep032_main	SHARE main job: I receive the recognition I deserve for my work.	X		
ep033_main	SHARE main job: Considering all my efforts and achievements, my salary is/earnings are adequate	X		
lowcontrol_ci_main	SHARE main job: =1 low control (separately calculated for each country)	X		
ERI_main	SHARE main job: Effort-reward imbalance (>1 poor quality of work)	X		
ERIi_main	SHARE main job: =1 poor quality of work	X		
ERIci_main	SHARE main job: =1 poor quality of work (separately calculated for each country)	X		
Biomarker				
maxgrip	Max. of grip strength measure	X	x	x
General Health				
ph006d1	Doctor told you had: heart attack	X	x	x
ph006d2	Doctor told you had: high blood pressure or hypertension	X	x	x
ph006d3	Doctor told you had: high blood cholesterol	X	x	
ph006d4	Doctor told you had: stroke	X	x	x
ph006d5	Doctor told you had: diabetes or high blood sugar	X	x	x
ph006d6	Doctor told you had: chronic lung disease	X	x	x
ph006d10	Doctor told you had: cancer	X	x	x
ph006d11	Doctor told you had: stomach or duodenal ulcer, peptic ulcer	X		
ph006d12	Doctor told you had: Parkinson disease	X	x	
ph006d13	Doctor told you had: cataracts	X	x	
ph006d14	Doctor told you had: hip fracture or femoral fracture	X	x	
ph006d15	Doctor told you had: other fractures	X		
ph006d16	Doctor told you had: alzheimer's disease, dementia, senility	X	x	x
ph006d18	Doctor told you had: other affective/emotional disorders	X	x	x
ph006d19	Doctor told you had: rheumatoid arthritis	X	x	x
ph006d20	Doctor told you had: osteoarthritis/other rheumatism	X	x	
illnesses_adult_ever	Sum (0-9) ever had illness (Adult)	X	x	x
ph061_	Health problem that limits paid work	X	x	x
sphus	Self-perceived health – us version	X	x	x
hs054_	number periods of ill health	X	x	
Mental Health				
eurod	Depression scale EURO-D - high is depressed	X		
eurod_lin1	Predicted value (linear Regression) for ELSA and HRS	X	x	x
cesd	CES-D Score		x	x
Limitations in activities of daily living				
ph049d1	Difficulties: dressing, including shoes and socks	X	x	x
ph049d2	Difficulties: walking across a room	X	x	x
ph049d3	Difficulties: bathing or showering	X	x	x
ph049d4	Difficulties: eating, cutting up food	X	x	x
ph049d5	Difficulties: getting in or out of bed	X	x	x
ph049d6	Difficulties: using the toilet, incl getting up or down	X	x	x
ph049d7	Difficulties: using a map in a strange place	X	x	x
ph049d8	Difficulties: preparing a hot meal	X	x	x
ph049d9	Difficulties: shopping for groceries	X	x	x

ph049d10	Difficulties: telephone calls	X	x	x
ph049d11	Difficulties: taking medications	X	x	x
ph049d12	Difficulties: doing work around the house or garden	X	x	
ph049d13	Difficulties: managing money	X	x	x
iadl	number of limitations with instrumental activities of daily living	X	x	x
adl	Number of limitations with activities of daily living	X	x	x
Life course history				
backpain_adult	adulthood illness: back pain (16+)	X	x	
arthr_adult	adulthood illness: arthritis... (16+)	X	x	
osteo_adult	adulthood illness: osteoporosis (16+)	X	x	
angina_adult	adulthood illness: angina or heart attack (16+)	X	x	
heart_adult	adulthood illness: other heart disease (16+)	X	x	
diab_adult	adulthood illness: diabetes or high blood sugar (16+)	X	x	
stroke_adult	adulthood illness: stroke (16+)	X	x	
asthma_adult	adulthood illness: asthma (16+)	X	x	
respiratory_adult	adulthood illness: respiratory problems (16+)	X	x	
headaches_adult	adulthood illness: severe headaches or migraines (16+)	X	x	
cancer_adult	adulthood illness: cancer or malignant tumour or leukaemia or lymphoma (16+)	X	x	
psych_adult	adulthood illness: Emotional, nervous, or psychiatric problem, incl. burnout (16+)	X	x	
fatigue_adult	adulthood illness: fatigue, e.g. with ME, MS (16+)	X	x	
eyesight_adult	adulthood illness: eyesight problems (16+)	X	x	
infectious_adult	adulthood illness: Infectious disease (16+)	X	x	
allergies_adult	adulthood illness: allergies (other than asthma) (16+)	X	x	
illnesses_adult_16	sum adulthood illnesses (16+) (0-16)	X	x	
infectious_ch	childhood illness: infectious disease	X	x	x
asthma_ch	childhood illness: asthma	X	x	x
respiratory_ch	childhood illness: respiratory problems	X	x	x
allergies_ch	childhood illness: allergies	X	x	x
ear_ch	childhood illness: ear problems	X		
headaches_ch	childhood illness: headaches or migraines	X	x	x
epilepsy_ch	childhood illness: epilepsy, fits or seizures	X	x	x
psych_ch	childhood illness: emotional, nervous, or psychiatric problem	X	x	x
fractures_ch	childhood illness: fractures	X		
diabetes_ch	childhood illness: diabetes or high blood sugar	X	x	x
heart_ch	childhood illness: heart trouble	X	x	x
cancer_ch	childhood illness: cancer (incl. leukaemia)	X	x	x
illnesses_ch	sum childhood illnesses	X	x	x
cs002	rooms when ten years old	X	x	
cs003	number of people living in household when ten	X	x	
cs008	number of books when ten	X	x	
cs010	relative position to others mathematically when ten	X		
Cognition				
cf003_	Date: day of month	X	x	x
cf004_	Date: month	X	x	x

cf005_	Date: year	X	x	x
cf006_	Date: day of the week	X	x	x
cf008tot	Ten words list learning first trial total	X	x	x
cf016tot	Ten words list learning delayed recall total	X	x	x

Table B3: List of variables where information needs to be merged from previous waves

Merged from previous waves				
Variable	Description	SHARE	ELSA	HRS
<u>Demographic</u>				
married	Is respondent married?	X		x
ever_married	Has respondent ever been married?	X	x	x
divorced	Is respondent divorced?	X		x
ever_divorced	Has respondent ever been divorced?	X	x	
widowed	Is respondent widowed?	X		x
ever_widowed	Has respondent ever been widowed?	X	x	x
<u>Education</u>				
dn041_	years of education	X	x	
educat	education category	X	x	
<u>Job</u>				
numberjobs				x
<u>General Health</u>				
ph006d1	Doctor told you had: heart attack		x	x
ph006d2	Doctor told you had: high blood pressure or hypertension		x	x
ph006d3	Doctor told you had: high blood cholesterol			
ph006d4	Doctor told you had: stroke		x	x
ph006d5	Doctor told you had: diabetes or high blood sugar		x	x
ph006d6	Doctor told you had: chronic lung disease		x	x
ph006d10	Doctor told you had: cancer		x	x
ph006d11	Doctor told you had: stomach or duodenal ulcer, peptic ulcer			
ph006d12	Doctor told you had: Parkinson disease			
ph006d13	Doctor told you had: cataracts			
ph006d14	Doctor told you had: hip fracture or femoral fracture			
ph006d15	Doctor told you had: other fractures			
ph006d16	Doctor told you had: alzheimer's disease, dementia, senility			x
ph006d18	Doctor told you had: other affective/emotional disorders		x	x
ph006d19	Doctor told you had: rheumatoid arthritis			x
ph006d20	Doctor told you had: osteoarthritis/other rheumatism			
<u>Childhood Illnesses</u>				
infectious_ch	childhood illness: infectious disease			x
asthma_ch	childhood illness: asthma			x
respiratory_ch	childhood illness: respiratory problems			x
allergies_ch	childhood illness: allergies			x
ear_ch	childhood illness: ear problems			x
headaches_ch	childhood illness: headaches or migraines			x
epilepsy_ch	childhood illness: epilepsy, fits or seizures			x
psych_ch	childhood illness: emotional, nervous, or psychiatric problem			x
fractures_ch	childhood illness: fractures			x

Table B4: List of variables including original variable names and data sources

Variable	SHARE Variables	SHARE Data source	ELSA Variables	ELSA Data source	HRS Variables	HRS Data source
Disability benefits						
di_receipt	ep071d4, ep071d5	sharew5_rel1-0-0_ep	iahdnsp, iahdnib, iahdnsd, iahdnaa, iahdndl, iahdnii, iahdn95, iahdnca, iahdnwd, iahdbc	wave_6_elsa_data_v2	r11isdi, r11issi, r11iwcmp	rndhrs_o
di_year	ep213_4, ep213_5	sharew5_rel1-0-0_ep	missing		dis1, radrecy1, radrecy2, radrecy3, radrecy4, radrecy5, radrecy6, radrecy7, radrecy8, radrecy9, radrecy10, radrecy11	rndhrs_o
Identifiers (merging…)						
Respondent identifier	mergeid	general	idauniq	General	hhidpn	general
Demographic						
country	country	general	just UK		just USA	
yrbirth	dn003_	sharew5_rel1-0-0_dn	indobyr	wave_6_elsa_data_v2	rabyear	rndhrs_o
age	dn002_, dn003_, int_month	sharew5_rel1-0-0_dn, sharew5_rel1-0-0_cv_r	indager	wave_6_elsa_data_v2	r11agey_e, rabyear, r11iwendy	rndhrs_o
gender	dn042_	sharew5_rel1-0-0_dn	indsex	wave_6_elsa_data_v2	ragender	rndhrs_o
married	wave 1,2,4,5: dn041_	sharew1_rel2-6-0_dn, sharew2_rel2-6-0_dn, sharew4_rel1-1-1_dn, sharew5_rel1-0-0_dn	dimar	wave_6_elsa_data_v2	r11mstath, r11mnev, r10mstath, r10mnev, r9mstath, r9mnev, r8mstath, r8mnev, r7mstath, r7mnev, r6mstath, r6mnev, r5mstath, r5mnev, r4mstath, r4mnev, r3mstath, r3mnev, r2mstath, r2mnev, r1mstath, r1mnev	rndhrs_o

ever_married	wave 1,2,4,5: dn041_ ; wave3: sl_rp002_, sl_rp002e_	sharew1_rel2-6-0_dn, sharew2_rel2-6-0_dn, sharew3_rel1_rp, sharew4_rel1-1-1_dn, sharew5_rel1-0-0_dn	wave 0: MARITALB, marital; wave 1,3,4,5: dimar; wave 2: DiMar; wave 6: dimar	wave 0: wave_0_common_variables_v2, wave_0_1998_data, wave_0_1999_data, wave_0_2001_data; wave 1: wave_1_core_data_v3; wave 2: wave_2_core_data_v4; wave 3: wave_3_elsa_data_v4´; wave 4: wave_4_elsa_data_v3; wave 5: wave_5_elsa_data_v4; wave 6: wave_6_elsa_data_v2;	r11mstath, r11mnev, r10mstath, r10mnev, r9mstath, r9mnev, r8mstath, r8mnev, r7mstath, r7mnev, r6mstath, r6mnev, r5mstath, r5mnev, r4mstath, r4mnev, r3mstath, r3mnev, r2mstath, r2mnev, r1mstath, r1mnev	rndhrs_o
divorced	wave 1,2,4,5: dn041_	sharew1_rel2-6-0_dn, sharew2_rel2-6-0_dn, sharew4_rel1-1-1_dn, sharew5_rel1-0-0_dn	dimar	wave_6_elsa_data_v2	r11mstath, r11mnev, r10mstath, r10mnev, r9mstath, r9mnev, r8mstath, r8mnev, r7mstath, r7mnev, r6mstath, r6mnev, r5mstath, r5mnev, r4mstath, r4mnev, r3mstath, r3mnev, r2mstath, r2mnev, r1mstath, r1mnev	rndhrs_o
ever_divorced	wave 1, 2, 4, 5: dn041_ ; wave3: sl_rp002e_, sl_rp013_1 - sl_rp013_4	sharew1_rel2-6-0_dn, sharew2_rel2-6-0_dn, sharew3_rel1_rp, sharew4_rel1-1-1_dn, sharew5_rel1-0-0_dn	wave 0: MARITALB, marital; wave 1,3,4,5: dimar; wave 2: DiMar; wave 6: dimar	wave 0: wave_0_common_variables_v2, wave_0_1998_data, wave_0_1999_data, wave_0_2001_data; wave 1: wave_1_core_data_v3; wave 2: wave_2_core_data_v4;	r11mstath, r11mnev, r10mstath, r10mnev, r9mstath, r9mnev, r8mstath, r8mnev, r7mstath, r7mnev, r6mstath,	rndhrs_o

				wave 3: wave_3_elsa_data_v4´; wave 4: wave_4_elsa_data_v3; wave 5: wave_5_elsa_data_v4; wave 6: wave_6_elsa_data_v2;	r6mnev, r5mstath, r5mnev, r4mstath, r4mnev, r3mstath, r3mnev, r2mstath, r2mnev, r1mstath, r1mnev	
widowed	wave 1, 2, 4, 5: dn041_	sharew1_rel2-6-0_dn, sharew2_rel2-6-0_dn, sharew4_rel1-1-1_dn, sharew5_rel1-0-0_dn	dimar	wave_6_elsa_data_v2	r11mstath, r11mnev, r10mstath, r10mnev, r9mstath, r9mnev, r8mstath, r8mnev, r7mstath, r7mnev, r6mstath, r6mnev, r5mstath, r5mnev, r4mstath, r4mnev, r3mstath, r3mnev, r2mstath, r2mnev, r1mstath, r1mnev	rndhrs_o
ever_widowed	wave 1, 2, 3, 4n041_	sharew1_rel2-6-0_dn, sharew2_rel2-6-0_dn, sharew4_rel1-1-1_dn, sharew5_rel1-0-0_dn	wave 0: MARITALB, marital; wave 1,3,4,5: dimar; wave 2: DiMar; wave 6: dimar	wave 0: wave_0_common_variables_v2, wave_0_1998_data, wave_0_1999_data, wave_0_2001_data; wave 1: wave_1_core_data_v3; wave 2: wave_2_core_data_v4; wave 3: wave_3_elsa_data_v4´; wave 4: wave_4_elsa_data_v3; wave 5: wave_5_elsa_data_v4; wave 6: wave_6_elsa_data_v2;	r11mstath, r11mnev, r10mstath, r10mnev, r9mstath, r9mnev, r8mstath, r8mnev, r7mstath, r7mnev, r6mstath, r6mnev, r5mstath, r5mnev, r4mstath, r4mnev, r3mstath, r3mnev, r2mstath, r2mnev,	rndhrs_o

					r1mstath, r1mnev	
Education						
dn041_<br><br>Collapsed at 14: 14+ because of ELSA	wave 2, 4, 5: dn041_, wave 1: iscedy_r	sharew1_rel2-6-0_gv_isced , sharew2_rel2-6-0_dn, sharew4_rel1-1-1_dn, sharew5_rel1-0-0_dn	wave 0: educend;<br>wave 1,3,4,5: fqend;<br>wave 2: FqEnd;<br>wave 6: fqend;	wave 0: wave_0_common_variables_v2, wave_0_1998_data, wave_0_1999_data, wave_0_2001_data;<br>wave 1: wave_1_core_data_v3;<br>wave 2: wave_2_core_data_v4;<br>wave 3: wave_3_elsa_data_v4;<br>wave 4: wave_4_elsa_data_v3;<br>wave 5: wave_5_elsa_data_v4;<br>wave 6: wave_6_elsa_data_v2;	raedyrs	rndhrs_o
educat	wave 1,2,4,5: isced 1997	sharew1_rel2-6-0_gv_isced, sharew2_rel2-6-0_gv_isced, sharew4_rel1-1-1_gv_isced, sharew5_rel1-0-0_gv_isced	wave 1,2,3,4,5,6,: edqual;	wave 1: wave_1_core_data_v3;<br>wave 2: wave_2_ifs_derived_variables;<br>wave 3: wave_3_elsa_data_v4;<br>wave 4: wave_4_elsa_data_v3;<br>wave 5: wave_5_elsa_data_v4;<br>wave 6: wave_6_ifs_derived_variables;	raedegrm, raeduc	rndhrs_o
Job						
numberjobs	Based on: year started job - sl_re011_1- sl_re011_20 in SHARELIFE (wave 3)	sharew3_rel1_re	wave 1: wpever;<br>wave 2: wpsjoby, wpllsy, wplljy, wplpey, wplpsy, wplpsy2, wplps3, wplpsy4, wplpsy5, wplpey2, wplpey3, wplpey4, wplpey5, wpever;<br>wave 3: rwjstyr, rwjstyr2-rwjstyr9, rwjsty10-rwjsty20, rwevw;<br>wave 4: wpsjoby, wplpey, wplpsy, wplpey2, wplpey3, wplpsy2, wplpsy3, wpever;	wave 1: wave_1_core_data_v3;<br>wave 2: wave_2_core_data_v4;<br>wave 3: wave_3_life_history_data;<br>wave 4: wave_4_elsa_data_v3;<br>wave 5: wave_5_elsa_data_v4;<br>wave 6: wave_6_elsa_data_v2;	r11jnjob, r10jnjob, r9jnjob, r8jnjob, r7jnjob, r6jnjob, r5jnjob, r4jnjob, r3jnjob, r2jnjob, r1jnjob	rndhrs_o

			wave 5: wpsjoby, wplpey, wplpsy, wplpey2, wplpey3, wplpey4, wplpey5, wplpsy2, wplpsy3, wplpsy4, wplpsy5, wpever; wave 6: wpsjoby, wplpsy, wplpsy2- wplpsy5, wpever			
working_gaps	Based on: sl_re033_1- sl_re033_17 SHARELIFE (wave 3)	sharew3_rel1_re	rwst4a- rwst4t, rwst1a- rwst1t, rwst2a- rwst2t, rwst3a- rwst3t, rwst5a- rwst5t, rwst6a- rwst6t, rwst7a- rwst7t, rwst8a- rwst8t, rwst9a- rwst9t, rwst95a- rwst95t, rwsti, rwsti2- rwsti20;	wave_3_life_history_data	Missing	
ep027_	ep027_	sharew5_rel1-0-0_ep	scworkb	wave_6_elsa_data_v2	nlb084b	h12f1a
ep028_	ep028_	sharew5_rel1-0-0_ep	scworkg	wave_6_elsa_data_v2	nlb084b	h12f1a
ep029_	ep029_	sharew5_rel1-0-0_ep	scworkh	wave_6_elsa_data_v2	nlb084h	h12f1a
ep030_	ep030_	sharew5_rel1-0-0_ep	scworki	wave_6_elsa_data_v2	nlb084i	h12f1a
ep031_	ep031_	sharew5_rel1-0-0_ep	scworkj	wave_6_elsa_data_v2	nlb084j	h12f1a
ep032_	ep032_	sharew5_rel1-0-0_ep	scworkc	wave_6_elsa_data_v2	nlb084c	h12f1a
ep033_	ep033_	sharew5_rel1-0-0_ep	scworkd	wave_6_elsa_data_v2	nlb084d	h12f1a
ep034_	ep034_	sharew5_rel1-0-0_ep	scworke	wave_6_elsa_data_v2	nlb084e	h12f1a
ep035_	ep035_	sharew5_rel1-0-0_ep	scworkf	wave_6_elsa_data_v2	nlb084f	h12f1a
lowcontrol_ci	ep029_ , ep030_, country	sharew5_rel1-0-0_ep	scworkh, scworki	wave_6_elsa_data_v2	nlb084h , nlb084i	h12f1a
ERI	ep027_, ep028_, ep031_, ep032_, ep033_, ep034_, ep035_,	sharew5_rel1-0-0_ep	scworkb , scworkg , scworkj, scworkc, scworkd, scworke, scworkf	wave_6_elsa_data_v2	nlb084b, nlb084b, nlb084j, nlb084c, nlb084d, nlb084e, nlb084f	h12f1a
ERIi	ERI	sharew5_rel1-0-0_ep	ERI	wave_6_elsa_data_v2	ERI	h12f1a
ERIci	ERI, country	sharew5_rel1-0-0_ep	ERI	wave_6_elsa_data_v2	ERI	h12f1a
ep027_main	SHARELIFE (wave 3): sl_wq002_	sharew3_rel1_wq	Missing		Missing	
ep028_main	SHARELIFE (wave 3): sl_wq004_	sharew3_rel1_wq	Missing		Missing	
ep029_main	SHARELIFE (wave 3): sl_wq007_	sharew3_rel1_wq	Missing		Missing	
ep030_main	SHARELIFE (wave 3): sl_wq008_	sharew3_rel1_wq	Missing		Missing	
ep031_main	SHARELIFE (wave 3): sl_wq011_	sharew3_rel1_wq	Missing		Missing	
ep032_main	SHARELIFE (wave 3): sl_wq009_	sharew3_rel1_wq	Missing		Missing	

ep033_main	SHARELIFE (wave 3): sl_wq010_	sharew3_rel1_wq	Missing		Missing	
lowcontrol_ci_main	ep029_ main, ep030_main, country	sharew3_rel1_wq	Missing		Missing	
ERI_main	ep031_main, ep032_main, ep033_main, ep034_main, ep035_main, ep027_main, ep028_main	sharew3_rel1_wq	Missing		Missing	
ERIi_main	ERI_main	sharew3_rel1_wq	Missing		Missing	
ERIci_main	ERI_main, country	sharew3_rel1_wq	Missing		Missing	
Biomarker						
maxgrip	maxgrip	sharew5_rel1-0-0_gv_health	mmgsd1, mmgsd2, mmgsdom	wave_6_elsa_nurse_data_v2	ni816, ni852, ni851, ni853	h12f1a
General Health						
ph006d1	ph006d1	sharew5_rel1-0-0_ph	wave 0: illsm1- illsm5 (=16);<br>wave 1,2,3,4,5: hefrac;<br>wave 6: hediami, hedacmi, hedawmi, heagb, henmmi, hedanmi, hediahf, hedashf, hedawmi, hedachf, heagc, hedanhf, hediahm, hedashm, hedawhm, hedachm, hedanhm, hediaar, hedasar, hedawhm, hedacar, hedanar, hedia95, hedasot, hedawot, hedacot, hedanot;	wave 0: wave_0_common_variables_v2, wave_0_1998_data, wave_0_1999_data, wave_0_2001_data;<br>wave 1: wave_1_core_data_v3;<br>wave 2: wave_2_core_data_v4;<br>wave 3: wave_3_elsa_data_v4;<br>wave 4: wave_4_elsa_data_v3;<br>wave 5: wave_5_elsa_data_v4;<br>wave 6: wave_6_elsa_data_v2;	r11hearte, r10hearte, r9hearte, r8hearte, r7hearte, r6hearte, r5hearte, r4hearte, r3hearte, r2hearte, r1hearte	rndhrs_o
ph006d2	ph006d2	sharew5_rel1-0-0_ph	wave 0: illsm1- illsm5 (=17);<br>wave 1,2,3,4,5: hefrac;<br>wave 6: hediabp, hedasbp, hedawbp, hedacbp, hedanbp;	wave 0: wave_0_common_variables_v2, wave_0_1998_data, wave_0_1999_data, wave_0_2001_data;<br>wave 1: wave_1_core_data_v3;<br>wave 2: wave_2_core_data_v4;<br>wave 3: wave_3_elsa_data_v4;<br>wave 4: wave_4_elsa_data_v3;<br>wave 5: wave_5_elsa_data_v4;<br>wave 6: wave_6_elsa_data_v2;	r11hibpe, r10hibpe, r9hibpe, r8hibpe, r7hibpe, r6hibpe, r5hibpe, r4hibpe, r3hibpe, r2hibpe, r1hibpe	rndhrs_o

ph006d3	ph006d3	sharew5_rel1-0-0_ph	wave 6: hediach, hedasch, hedawch, hedacch, hedanch;	wave 6: wave_6_elsa_data_v2;	missing	
ph006d4	ph006d4	sharew5_rel1-0-0_ph	wave 0: illsm1- illsm5 (=15); wave 1,2,3,4,5: hefrac; wav 6: hediast, hedawst, hedacst, heage, henmst, hedanst,	wave 0: wave_0_common_variables_v2, wave_0_1998_data, wave_0_1999_data, wave_0_2001_data; wave 1: wave_1_core_data_v3; wave 2: wave_2_core_data_v4; wave 3: wave_3_elsa_data_v4; wave 4: wave_4_elsa_data_v3; wave 5: wave_5_elsa_data_v4; wave 6: wave_6_elsa_data_v2;	r11stroke, r10stroke, r9stroke, r8stroke, r7stroke, r6stroke, r5stroke, r4stroke, r3stroke, r2stroke, r1stroke	rndhrs_o
ph006d5	ph006d5	sharew5_rel1-0-0_ph	wave 0: illsm1- illsm5 (=2); wave 1,2,3,4,5: hefrac; wave 6: hediadi, hedawdi, hedacdi, hedandi,	wave_0_common_variables_v2, wave_0_1998_data, wave_0_1999_data, wave_0_2001_data; wave 1: wave_1_core_data_v3; wave 2: wave_2_core_data_v4; wave 3: wave_3_elsa_data_v4; wave 4: wave_4_elsa_data_v3; wave 5: wave_5_elsa_data_v4; wave 6: wave_6_elsa_data_v2;	r11diabe, r10diabe, r9diabe, r8diabe, r7diabe, r6diabe, r5diabe, r4diabe, r3diabe, r2diabe, r1diabe	rndhrs_o
ph006d6	ph006d6	sharew5_rel1-0-0_ph	wave 0: illsm1- illsm5 (=22); wave 1,2,3,4,5: hefrac; wave 6: hediblu, hedblu, hedbwlu, hedbdlu, hedbmlu;	wave_0_common_variables_v2, wave_0_1998_data, wave_0_1999_data, wave_0_2001_data; wave 1: wave_1_core_data_v3; wave 2: wave_2_core_data_v4; wave 3: wave_3_elsa_data_v4; wave 4: wave_4_elsa_data_v3;	r11lunge, r10lunge, r9lunge, r8lunge, r7lunge, r6lunge, r5lunge, r4lunge, r3lunge, r2lunge, r1lunge	rndhrs_o

				wave 5: wave_5_elsa_data_v4; wave 6: wave_6_elsa_data_v2;		
ph006d10	ph006d10	sharew5_rel1-0-0_ph	wave 0: illsm1- illsm5 (=1); wave 1,2,3,4,5: hefrac; wave 6: hedibca, hedbsca, hedbwca, hedbdca, heagg, hedbmca;	wave_0_common_variables_v2, wave_0_1998_data, wave_0_1999_data, wave_0_2001_data; wave 1: wave_1_core_data_v3; wave 2: wave_2_core_data_v4; wave 3: wave_3_elsa_data_v4; wave 4: wave_4_elsa_data_v3; wave 5: wave_5_elsa_data_v4; wave 6: wave_6_elsa_data_v2;	r11cancre, r10cancre, r9cancre, r8cancre, r7cancre, r6cancre, r5cancre, r4cancre, r3cancre, r2cancre, r1cancre	rndhrs_o
ph006d11	ph006d11	sharew5_rel1-0-0_ph	missing		missing	
ph006d12	ph006d12	sharew5_rel1-0-0_ph	wave 6: hedibpd, hedbspd, hedbwpd, hedbdpd, heprk, hedbmpd;	wave 6: wave_6_elsa_data_v2;	missing	
ph006d13	ph006d13	sharew5_rel1-0-0_ph	wave 6: heoptca, heopsca, heopfca, heopcca, heopnca;	wave 6: wave_6_elsa_data_v2;	missing	
ph006d14	ph006d14	sharew5_rel1-0-0_ph	wave 1,2,3,4,5,6: hefrac;	wave 1: wave_1_core_data_v3; wave 2: wave_2_core_data_v4; wave 3: wave_3_elsa_data_v4; wave 4: wave_4_elsa_data_v3; wave 5: wave_5_elsa_data_v4; wave 6: wave_6_elsa_data_v2;	missing	
ph006d15	ph006d15	sharew5_rel1-0-0_ph	missing		missing	
ph006d16	ph006d16	sharew5_rel1-0-0_ph	wave 6: hedibad, hedbwad, hedbdad, heagi, hedbmad, hedibde, hedbsde, hedbwad¸ hedbdde, heagj, hedbmde;	wave 6: wave_6_elsa_data_v2;	r11alzhe, r10alzhe, r9alzhe, r8alzhe, r7alzhe, r6alzhe, r5alzhe, r4alzhe, r3alzhe,	rndhrs_o

					r2alzhe, r1alzhe	
ph006d18	ph006d18	sharew5_rel1-0-0_ph	wave 0: illsm1- illsm5 (=4); wave 6: hedibps, hedbwps, hedbdps, heagh, hedbmps, hepsyha, hepsyan¸ hepsyde, hepsyem, hepsysc, hepsyps, hepsymo, hepsyma, hepsy95, heyrc;	wave 0: wave_0_common_variables_v2, wave_0_1998_data, wave_0_1999_data, wave_0_2001_data; wave 6: wave_6_elsa_data_v2;	r11psyche, r10psyche, r9psyche, r8psyche, r7psyche, r6psyche, r5psyche, r4psyche, r3psyche, r2psyche, r1psyche	rndhrs_o
ph006d19	ph006d19	sharew5_rel1-0-0_ph	wave 6: hedibar, hedbsar, hedbwar, hedbdar, heagf, hedbmar, heartra;	wave 6: wave_6_elsa_data_v2;	r11arthre, r10arthre, r9arthre, r8arthre, r7arthre, r6arthre, r5arthre, r4arthre, r3arthre, r2arthre, r1arthre	rndhrs_o
ph006d20	ph006d20	sharew5_rel1-0-0_ph	wave 6: heartoa;	wave 6: wave_6_elsa_data_v2;	missing	
illnesses_adult_ever	Sum of ph006d1, ph006d2, ph006d4, ph006d5, ph006d6 , ph006d10, ph006d16, ph006d18, ph006d19	sharew5_rel1-0-0_ph	Sum of ph006d1, ph006d2, ph006d4, ph006d5, ph006d6 , ph006d10, ph006d16, ph006d18, ph006d19	wave 6: wave_6_elsa_data_v2;	Sum of ph006d1, ph006d2, ph006d4, ph006d5, ph006d6 , ph006d10, ph006d16, ph006d18, ph006d19	rndhrs_o
ph061_	ph061_	sharew5_rel1-0-0_ph	helwk	wave_6_elsa_data_v2	r11hlthlm	rndhrs_o
sphus	sphus (ph003)	sharew5_rel1-0-0_gv_health	hehelf	wave_6_elsa_data_v2	r11shlt	rndhrs_o
hs054_	hs054_ SHARELIFE (wave 3)	sharew3_rel1_hs	rhpbb	wave_3_life_history_data		
Mental Health						
eurod	eurod	sharew5_rel1-0-0_gv_health	Missing: see eurod_lin1		Missing: see eurod_lin1	
eurod_lin1	eurod	Prediction rule via linear Regression	Prediction: cesd1, cesd2, cesd3, cesd4, cesd5, cesd6, cesd7, cesd8, age, age2, age3, gender, sphus		Prediction: cesd1, cesd2, cesd3, cesd4, cesd5, cesd6, cesd7, cesd8, age, age2, age3, gender, sphus	

cesd	wave 1: q4_a, q4_b, q4_c, q4_d, q4_e, q4_g, q4_h, q4_j;	sharew1_rel2-6-0_dropoff	psceda, pscedb, pscedc, pscedd, pscede, pscedf, pscedg, pscedh	wave 6: wave_6_elsa_data_v2	r11depres, r11effort, r11sleepr, r11whappy, r11flone, r11enlife, r11fsad, r11going	rndhrs_o
cesd_lin1	Prediction based on wave 1: eurod, age, age2, age3, gender , sphus	Prediction rule via linear Regression	cesd		cesd	
Limitations in activities of daily living						
ph049d1	ph049d1	sharew5_rel1-0-0_ph	headldr	wave_6_elsa_data_v2	r11dress	rndhrs_o
ph049d2	ph049d2	sharew5_rel1-0-0_ph	headlwa	wave_6_elsa_data_v2	r11walkr	rndhrs_o
ph049d3	ph049d3	sharew5_rel1-0-0_ph	headlba	wave_6_elsa_data_v2	r11bath	rndhrs_o
ph049d4	ph049d4	sharew5_rel1-0-0_ph	headlea	wave_6_elsa_data_v2	r11eat	rndhrs_o
ph049d5	ph049d5	sharew5_rel1-0-0_ph	headlbe	wave_6_elsa_data_v2	r11bed	rndhrs_o
ph049d6	ph049d6	sharew5_rel1-0-0_ph	headlwc	wave_6_elsa_data_v2	r11toilt	rndhrs_o
ph049d7	ph049d7	sharew5_rel1-0-0_ph	headlma	wave_6_elsa_data_v2	r11mapa	rndhrs_o
ph049d8	ph049d8	sharew5_rel1-0-0_ph	headlpr	wave_6_elsa_data_v2	r11meals	rndhrs_o
ph049d9	ph049d9	sharew5_rel1-0-0_ph	headlsh	wave_6_elsa_data_v2	r11shop	rndhrs_o
ph049d10	ph049d10	sharew5_rel1-0-0_ph	headlph	wave_6_elsa_data_v2	r11phone	rndhrs_o
ph049d11	ph049d11	sharew5_rel1-0-0_ph	headlme	wave_6_elsa_data_v2	r11meds	rndhrs_o
ph049d12	ph049d12	sharew5_rel1-0-0_ph	headlho	wave_6_elsa_data_v2	missing	
ph049d13	ph049d13	sharew5_rel1-0-0_ph	headlmo	wave_6_elsa_data_v2	r11money	rndhrs_o
iadl	ph049d7, ph049d8, ph049d9, ph049d10, ph049d11, ph049d13	sharew5_rel1-0-0_ph	headlma, headlpr, headlsh, headlph, headlme, headlmo	wave_6_elsa_data_v2	r11mapa, r11meals, r11shop, r11phone, r11meds, r11money	rndhrs_o
adl	ph049d1, ph049d2, ph049d3, ph049d4, ph049d5, ph049d6	sharew5_rel1-0-0_gv_health	headldr, headlwa, headlba, headlea, headlbe, headlwc,	wave_6_elsa_data_v2	r11dress, r11walkr, r11bath, r11eat, r11bed, r11toilt	rndhrs_o
Life course history						
backpain_adult	SHARELIFE (wave 3): hs055d1_1, hs055d1_2, hs055d1_3	sharew3_rel1_hs	rhpbc1	wave_3_life_history_data	Missing	
arthr_adult	SHARELIFE (wave 3): hs055d2_1, hs055d2_2, hs055d2_3	sharew3_rel1_hs	rhpbc2	wave_3_life_history_data	Missing	
osteo_adult	SHARELIFE (wave 3): hs055d3_1, hs055d3_2, hs055d3_3	sharew3_rel1_hs	rhpbc3	wave_3_life_history_data	Missing	
angina_adult	SHARELIFE (wave 3): hs055d4_1, hs055d4_2, hs055d4_3	sharew3_rel1_hs	rhpbc4	wave_3_life_history_data	Missing	
heart_adult	SHARELIFE (wave 3): hs055d5_1, hs055d5_2, hs055d5_3	sharew3_rel1_hs	rhpbc5	wave_3_life_history_data	Missing	
diab_adult	SHARELIFE (wave 3): hs055d6_1, hs055d6_2, hs055d6_3	sharew3_rel1_hs	rhpbc6	wave_3_life_history_data	Missing	

stroke_adult	SHARELIFE (wave 3): hs055d7_1, hs055d7_2, hs055d7_3	sharew3_rel1_hs	rhpbc7	wave_3_life_history_data	Missing	
asthma_adult	SHARELIFE (wave 3): hs055d8_1, hs055d8_2, hs055d8_3	sharew3_rel1_hs	rhpbc8	wave_3_life_history_data	Missing	
respiratory_adult	SHARELIFE (wave 3): hs055d9_1, hs055d9_2, hs055d9_3	sharew3_rel1_hs	rhpbc9	wave_3_life_history_data	Missing	
headaches_adult	SHARELIFE (wave 3): hs055d11_1, hs055d11_2, hs055d11_3	sharew3_rel1_hs	rhpbc10	wave_3_life_history_data	Missing	
cancer_adult	SHARELIFE (wave 3): hs056d1_1, hs056d1_2, hs056d1_3, hs056d2_1, hs056d2_2, hs056d2_3,	sharew3_rel1_hs	rhpbx1, rhpbx2	wave_3_life_history_data	Missing	
psych_adult	SHARELIFE (wave 3): hs056d3_1, hs056d3_2, hs056d3_3,	sharew3_rel1_hs	rhpbx3	wave_3_life_history_data	Missing	
fatigue_adult	SHARELIFE (wave 3): hs056d4_1, hs056d4_2, hs056d4_3,	sharew3_rel1_hs	rhpbx4	wave_3_life_history_data	Missing	
eyesight_adult	SHARELIFE (wave 3): hs056d6_1, hs056d6_2, hs056d6_3,	sharew3_rel1_hs	rhpbx6	wave_3_life_history_data	Missing	
infectious_adult	SHARELIFE (wave 3): hs056d7_1, hs056d7_2, hs056d7_3,	sharew3_rel1_hs	rhpbx7	wave_3_life_history_data	Missing	
allergies_adult	SHARELIFE (wave 3): hs056d8_1, hs056d8_2, hs056d8_3,	sharew3_rel1_hs	rhpbx8	wave_3_life_history_data	Missing	
illnesses_adult_16	Sum of adulthood illnesses16+ listed above	sharew3_rel1_hs	Sum of adulthood illnesses16+ listed above	wave_3_life_history_data	Missing	
infectious_ch	SHARELIFE (wave 3): hs008d1, hs008d2; wave 5: mc012d1, mc012d2	sharew3_rel1_hs, sharew5_rel1-0-0_mc	rhcig1	wave_3_life_history_data	wave 9: lb100, lb101, lb102, lb125m1m, lb125m2m, lb125m3m, lb124;<br>wave 10: mb100, mb101, mb102, mb125m1m, mb125m2m, mb125m3m, mb124;<br>wave 11: nb100, nb101, nb102, nb125m1m, nb125m2m, nb125m3m, nb124;	h08f2a, hd10f5c, h12f1a.dta
asthma_ch	SHARELIFE (wave 3): hs008d3; wave 5: mc012d3	sharew3_rel1_hs, sharew5_rel1-0-0_mc	rhcig3	wave_3_life_history_data	wave 9: lb105;<br>wave 10: mb105;	h08f2a, hd10f5c, h12f1a.dta

					wave 11: nb105;	
respiratory_ch	SHARELIFE (wave 3): hs008d4; wave 5: mc012d4	sharew3_rel1_hs, sharew5_rel1-0-0_mc	rhcig5	wave_3_life_history_data	wave 9: lb107, lb125m1m, lb125m2m, lb125m3m, lb124; wave 10: mb107, mb125m1m, mb125m2m, mb125m3m, mb124; wave 11: nb107, nb125m1m, nb125m2m, nb125m3m, nb124;	h08f2a, hd10f5c, h12f1a.dta
allergies_ch	SHARELIFE (wave 3): hs008d5; wave 5: mc012d5	sharew3_rel1_hs, sharew5_rel1-0-0_mc	rhcig4	wave_3_life_history_data	wave 9: lb109; wave 10: mb109; wave 11: nb109;	h08f2a, hd10f5c, h12f1a.dta
ear_ch	SHARELIFE (wave 3): hs008d8; wave 5: mc012d8	sharew3_rel1_hs, sharew5_rel1-0-0_mc	rhcig6	wave_3_life_history_data	wave 9: lb111; wave 10: mb111; wave 11: nb111;	h08f2a, hd10f5c, h12f1a.dta
headaches_ch	SHARELIFE (wave 3): hs009d1; wave 5: mc013d1	sharew3_rel1_hs, sharew5_rel1-0-0_mc	rhcig7	wave_3_life_history_data	wave 9: lb113; wave 10: mb113; wave 11: nb113;	h08f2a, hd10f5c, h12f1a.dta
epilepsy_ch	SHARELIFE (wave 3): hs009d2; wave 5: mc013d2	sharew3_rel1_hs, sharew5_rel1-0-0_mc	rhcig8	wave_3_life_history_data	wave 9: lb112; wave 10: mb112; wave 11: nb112;	h08f2a, hd10f5c, h12f1a.dta
psych_ch	SHARELIFE (wave 3): hs009d3; wave 5: mc013d3	sharew3_rel1_hs, sharew5_rel1-0-0_mc	rhcig9	wave_3_life_history_data	wave 9: lb116, lb118, lb125m1m, lb125m2m, lb125m3m, lb124; wave 10: mb116, mb118, mb125m1m, mb125m2m, mb125m3m, mb124; wave 11: nb116, nb118, nb125m1m, nb125m2m, nb125m3m, nb124;	h08f2a, hd10f5c, h12f1a.dta
fractures_ch	SHARELIFE (wave 3): hs009d4; wave 5: mc013d4	sharew3_rel1_hs, sharew5_rel1-0-0_mc	rhcig2	wave_3_life_history_data	wave 9: lb125m1m, lb125m2m, lb125m3m, lb124; wave 10: mb125m1m, mb125m2m, mb125m3m, mb124; wave 11: nb125m1m, nb125m2m, nb125m3m, nb124;	h08f2a, hd10f5c, h12f1a.dta
diabetes_ch	SHARELIFE (wave 3): hs009d6; wave 5: mc013d6	sharew3_rel1_hs, sharew5_rel1-0-0_mc	rhcig11	wave_3_life_history_data	wave 9: lb106; wave 10: mb106; wave 11: nb106;	h08f2a, hd10f5c, h12f1a.dta
heart_ch	SHARELIFE (wave 3): hs009d7; wave 5: mc013d7	sharew3_rel1_hs, sharew5_rel1-0-0_mc	rhcig12	wave_3_life_history_data	wave 9: lb110; wave 10: mb110; wave 11: nb110;	h08f2a, hd10f5c, h12f1a.dta

cancer_ch	SHARELIFE (wave 3): hs009d8, hs009d9; wave 5: mc013d8, mc013d9	sharew3_rel1_hs, sharew5_rel1-0-0_mc	rhcig13, rhcig14	wave_3_life_history_data	wave 9: lb125m1m, lb125m2m, lb125m3m, lb124; wave 10: mb125m1m, mb125m2m, mb125m3m, mb124; wave 11: nb125m1m, nb125m2m, nb125m3m, nb124;	h08f2a, hd10f5c, h12f1a.dta
illnesses_ch	Sum of childhood illnesses listed above	sharew3_rel1_hs, sharew5_rel1-0-0_mc	Sum of childhood illnesses listed above	wave_3_life_history_data	Sum of childhood illnesses listed above	h08f2a, hd10f5c, h12f1a.dta
cs002	SHARELIFE (wave 3): cs002 & wave 5: mc003_	sharew3_rel1_cs,	raroo	wave_3_life_history_data	Missing	
cs003	SHARELIFE (wave 3): cs003 & wave 5: mc004_	sharew3_rel1_cs,	rapeo	wave_3_life_history_data	Missing	
cs008	SHARELIFE (wave 3): cs008 & wave 5: mc005_	sharew3_rel1_cs,	rabks	wave_3_life_history_data	Missing	
cs010	SHARELIFE (wave 3): cs010 & wave 5: mc006_	sharew3_rel1_cs,	missing		Missing	
Cognition						
cf003_	cf003_	sharew5_rel1-0-0_cf	cfdatd	wave_6_elsa_data_v2	r11dy	rndhrs_o
cf004_	cf004_	sharew5_rel1-0-0_cf	cfdatm	wave_6_elsa_data_v2	r11mo	rndhrs_o
cf005_	cf005_	sharew5_rel1-0-0_cf	cfdaty	wave_6_elsa_data_v2	r11yr	rndhrs_o
cf006_	cf006_	sharew5_rel1-0-0_cf	cfday	wave_6_elsa_data_v2	r11dw	rndhrs_o
cf008tot	cf008tot	sharew5_rel1-0-0_gv_health	cflisen	wave_6_elsa_data_v2	r11imr	rndhrs_o
cf016tot	cf016tot	sharew5_rel1-0-0_gv_health	cflisd	wave_6_elsa_data_v2	r11dlrc	rndhrs_o
